# Bimetallic Nanocrystals: Structure, Controllable Synthesis and Applications in Catalysis, Energy and Sensing

**DOI:** 10.3390/nano11081926

**Published:** 2021-07-26

**Authors:** Gaojie Li, Wenshuang Zhang, Na Luo, Zhenggang Xue, Qingmin Hu, Wen Zeng, Jiaqiang Xu

**Affiliations:** 1 NEST Lab, Department of Physics, College of Science, Shanghai University, Shanghai 200444, China; nihaoluona@163.com (N.L.); xuezheng@mail.ustc.edu.cn (Z.X.); hqingm@126.com (Q.H.); 2 School of Physics and Engineering, Henan University of Science and Technology, Luoyang 471023, China; 3 NEST Lab, Department of Chemistry, College of Science, Shanghai University, Shanghai 200444, China; wenshuangzhang@foxmail.com; 4 School of Materials Science and Engineering, Chongqing University, Chongqing 400044, China

**Keywords:** bimetallic nanocrystals, controllable synthesis, catalytic applications, gas sensing application, Bio-detection applications

## Abstract

In recent years, bimetallic nanocrystals have attracted great interest from many researchers. Bimetallic nanocrystals are expected to exhibit improved physical and chemical properties due to the synergistic effect between the two metals, not just a combination of two monometallic properties. More importantly, the properties of bimetallic nanocrystals are significantly affected by their morphology, structure, and atomic arrangement. Reasonable regulation of these parameters of nanocrystals can effectively control their properties and enhance their practicality in a given application. This review summarizes some recent research progress in the controlled synthesis of shape, composition and structure, as well as some important applications of bimetallic nanocrystals. We first give a brief introduction to the development of bimetals, followed by the architectural diversity of bimetallic nanocrystals. The most commonly used and typical synthesis methods are also summarized, and the possible morphologies under different conditions are also discussed. Finally, we discuss the composition-dependent and shape-dependent properties of bimetals in terms of highlighting applications such as catalysis, energy conversion, gas sensing and bio-detection applications.

## 1. Introduction

Crystals with at least one dimension at the nanometer level (1~100 nm) are known as nanocrystals. At the nanoscale, materials exhibit fascinating physical and chemical properties due to quantum size effect [1,2]. For instance, bulk gold has no activity for CO oxidation at low temperatures, while nanoscale gold has high catalytic activity [3]. Therefore, the controlled synthesis and application of metal nanocrystals have always been a research hotspot in the field of nanochemistry. The composition and structure of noble metals are relatively simple, and they have been used in many applications such as catalysis [4,5,6], electronics [7], and information storage [8] and so on. However, single metal nanocrystals have some shortcomings that are difficult to overcome in the application process. For example, although Pt catalyst has high catalytic activity for CO oxidation, it is easily poisoned. In addition, the amount of noble metals on earth are very low, and the reserves are extremely limited. Therefore, bimetallic nanocrystals have drawn the attention of a large number of researchers because bimetal exhibits not only a combination of two monometallic properties, but also some new properties due to synergistic effects [9,10,11,12,13].

**Figure 1:**
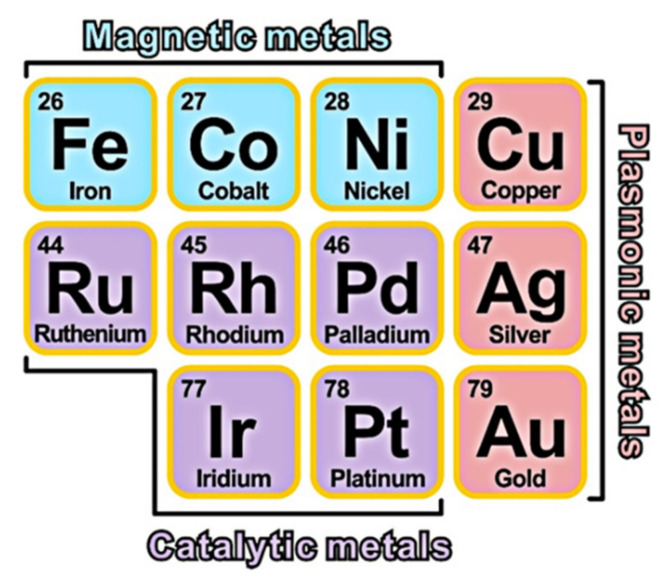
A part of the periodic table of elements and their own inherent properties. Reproduced with permission from [16]. Copyright American Chemical Society, 2016.

As shown in Figure 1, metals have their own inherent properties, such as magnetic metal (Fe, Co, Ni), plasmonic metal (Cu, Au, Ag), and catalytic metal (Pd, Pt, Rh, Ru, Ir). However, some noble metals are relatively rare on earth, and the price is quite high. In the process of preparing catalysts, the commonly used precious metal precursors are chlorides or nitrates. Taking chlorides as an example, the order of the price of precious metals is RhCl_3_ > IrCl_3_ > AuCl > PdCl_2_ > PtCl_2_ > RuCl_3_. The price of the cheapest RuCl_3_ is about 150-fold that of NiCl_2_·6H_2_O, and 70-folds than that of CuCl_2_·2H_2_O. In addition, the properties of a single metal are often not perfect. Nanocatalysts composed of two different metals have excellent catalytic performance and relatively low cost, and are becoming a frontier basic subject of interdisciplinary research in inorganic chemistry, nanoscience, and catalytic chemistry. Since bimetals have a two-component common energy band, by changing the composition of the alloy, the electronic structure and geometric configuration of the particle surface can be continuously changed by adjusting the distance between the d-band position of the metal and the Fermi energy level, It can change the adsorption capacity of nanoparticles for reactants and products, and then affect the activity, selectivity and stability of the catalyst [14]. Many works in the literature have proved that PtNi catalyst exhibited much higher catalytic performance than pure Pt for the oxygen reduction reaction (ORR) in fuel cells. For example, Zhao and co-workers reported that intermetallic PtNi catalyst exhibited remarkably enhanced ORR activity in comparison to the disordered PtNi and commercial Pt/C, and the durability also was improved by N doping [15].

The physicochemical properties of bimetallic nanocrystals are highly related to the spatial arrangement, atomic ordering and proportion of the different types of atoms. Therefore, the physicochemical properties of bimetallic nanocrystals are often highly adjustable and can be precisely designed by changing composition and optimizing structure according to need. Thus, a large amount of effort has been focused on synthesizing bimetallic nanocrystals with well-defined facets (e.g., cube, tetrahedron, octahedron, rhombic dodecahedron, and hexagonal plates) and special structure (core-shell, alloy and heterostructure). Owing to the efforts of many scientists all over the world in recent years [16,17,18,19,20], many effective synthetic strategies have been developed, which are able to prepare a variety of bimetallic nanocrystals with different structures and components. The properties of bimetallic nanocatalysts including activity, selectivity and stability, have been proved to be not only related to the composition and size of the particles, but also related to the types of nanostructures. Huang and co-workers demonstrated that PdPt nanocube exhibited higher activity towards the hydrogenation of nitrobenzene than PdPt tetrahedral, and Pt_51_Pd_49_ is higher than others [21]. Obviously, it is an urgent task in this field to develop some liquid-phase synthesis methods that can control nanocrystals with specific size, composition and morphology, and it has also become a frontier topic in the field of bimetallic catalysis.

Due to the excellent works of many research groups, some synthesis methods were optimized and new synthetic strategies were designed. In addition, the application fields of bimetallic nanocrystals have also been expanded. This review mainly covers the most recent developments of typical structure, synthesis methods, and the application in many important fields. In this review, there are 74 references to be cited for the periods 2011–2015 and 97 references for the periods 2016–2020. Bimetals containing only noble metals are involved in 72 references, and bimetals containing at least one non-noble metal (iron, nickel, cobalt, copper, tin and titanium) have 82 references, in which the most widely studied bimetal is PtNi (22 references). Transition metal Ni has been proved an excellent additive in Pt-based catalysts, and Pt–Ni alloy nanocrystals are effective catalysts for electrocatalysis and heterogeneous catalysis due to the adjustable electronic structure, morphology and stoichiometry. In addition, it is worth noting that some related review articles of bimetals have been published about liquid-phase synthesis [17], shape-controlled synthesis [22], magnetic and optical properties and applications [16], magnetic and optical properties for biological applications [23], SPR and LSPR properties for photo catalysis [24] and sensing [25], and theory to applications [19]. These reviews fully illustrate the importance of the research field of bimetallic nanocrystals. In addition, we try our best to avoid overlap in preparing this review. Compared to other related reviews, this article will focus on controllable synthesis of typical bimetallic nanostructures and highlighting the most recent advance in some hot fields such as catalysis, energy conversion, sensing and bio-detection applications by selecting the represent articles.

## 2. The Structures of Bimetallic Nanocrystals

**Figure 2:**
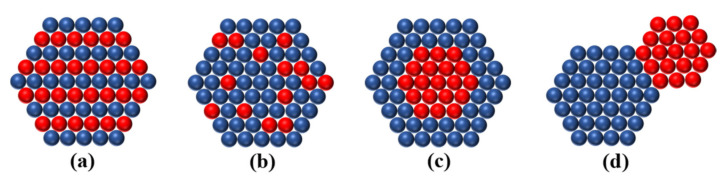
Bimetallic nanostructures: (**a**) intermetallic alloy, (**b**) solid solution alloy, (**c**) core-shell, and (**d**) heterostructure.

Despite only two elements, the architecture of bimetallic nanocrystals are complicated and multiform. According to the atomic ordering and configuration of two different metals, bimetallic nanostructures can be classified into three main types: alloyed structures (intermetallic and solid solution) and core-shell and heterostructure, as displayed in Figure 2. Due to the different raw materials and synthesis conditions, these nanostructures can also expose various crystal faces, including low Miller index surface, high Miller index step surface or their mixed crystal faces. In addition, the structure of bimetallic nanocrystals can be also divided into 0-D, 1-D and 2-D structures according to the three-dimensional extension of nanocrystals, as shown in Table 1. The zero-dimensional structure means that the expansion of the crystal in the three-dimensional direction is the same order of magnitude, mainly including regular polyhedrons and Wulff polyhedrons. One-dimensional structure refers to that the extension length of the crystal in one dimension is much greater than the extension length of the other two dimensions, mainly includes nanowires, nanorods, and so on. A two-dimensional structure implies that the stretched length of crystals in two dimensions is much greater than that of another dimension, mainly including nanoplates, nanosheets, and nanobelts etc. In addition, many derivative structures (core-frame, nanoboxes and nanocages) are reported in the literature.

### 2.1. Alloyed Structure

It has been proved that alloyed materials often exhibit unique physicochemical properties, not just the simple addition of the intrinsic properties of two distinct metals [49,50,51]. Alloyed structures of bimetallic nanocrystals are homogeneous mixtures with specific geometric arrangement of atoms, and metal bonds are formed between metal atoms. On the basis of atomic ordering, an alloyed structure can be divided into intermetallic compound and solid solution. The components in intermetallic compound alloys are bonded to each other to form compounds, so they have both long-range atomic order and definite atomic ratio. For a solid solution alloy, the components dissolve each other and the metal atoms randomly and thoroughly mixed. In the powder XRD pattern, the characteristic diffraction peak of single metal will disappear, and the alloy diffraction peak different from the two pure metal components will appear. The classification is highly significant because solid solution and intermetallic compounds often exhibit distinct performance even though they have the same elemental composition and stoichiometry.

For preparing the solid solution alloy, the most commonly used method is based on co-reduction (see Section 3.1). Although this method could effectively control the atomic ratio of products, it does not perform well in controlling the morphology and atomic order of nanocrystals [16]. The alloy structure of two metals (C and D) will be favored if three factors can be met: (i) relative strengths of C-D bond is higher than both the C-C and D-D bonds; (ii) similar surface energies of the individual bulk metals (C and D); and (iii) approximate atomic sizes and similar crystal structure [19]. It has been well proved that the atomic arrangement in nanoalloys strictly depends on the balance of the factors outlined above, and is also related to the experimental conditions and synthetic strategies.

Some bimetallic systems might tend to form new crystalline structures distinct from a single metal, and exhibit strict atomic arrangements rather than random mixing, such as PtCu_3_ [52], Pt_3_Ti [53], Pt_3_Sn [54], Ni_3_Sn_2_ [55], and PtFe [56]. Thermodynamically, the final atomic arrangements in bimetal can be predicted by the change in Gibbs free energy upon mixing (ΔG_mix_) under a certain condition. For an ideal binary system, ΔG_mix_ can be expressed by [57]

ΔG_mix_ = ΔH_mix_ − TΔS_mix_ (1)

where T is reaction temperature, ΔH_mix_ and ΔS_mix_ are the changes of enthalpy and entropy before and after the reaction, respectively. In principle, the more negative the ΔG_mix_, the easier it is for two metal atoms to be mutually soluble. In most case, however, intermetallic compounds are difficult to obtain by a liquid method, which tends to produce solid solution alloys with disordered atomic arrangements. Thus, in order to obtain intermetallic compounds from alloys, some post-treatment processes (high temperature heat treatment under inert gas protection) are often needed [53,58,59]. Intermetallic compounds often exhibit improved catalytic and magnetic properties over that of alloys due to the precise control of composition and surface atomic arrangement [60,61]. Hideki and co-workers prepared atomically disordered Pt_3_Ti nanoparticles by co-reduction method, and atomically disordered Pt_3_Ti nanoparticles could transfer into atomically ordered Pt_3_Ti nanoparticles by annealing in vacuum at 600 °C. Both materials were used as the electrocatalyst for fuel cells, and the results showed that Pt_3_Ti NPs with highly ordered atomic arrangements exhibited higher current densities for formic acid oxidation than that of atomically disordered Pt_3_Ti NPs [53]. Considering the magnetic properties, intermetallic compounds consisting of one 3d metal (such as Fe, Co, Ni) and one 4d or 5d metal (such as Pd, Pt) have been distinguished, which exhibited excellent performance in the field of information storage [16]. Above all, bimetallic nanocrystals composited of intermetallic compounds have broad prospects in catalytic, energy conversion and storage, and sensing applications.

### 2.2. Core-Shell Structure

**Figure 3:**
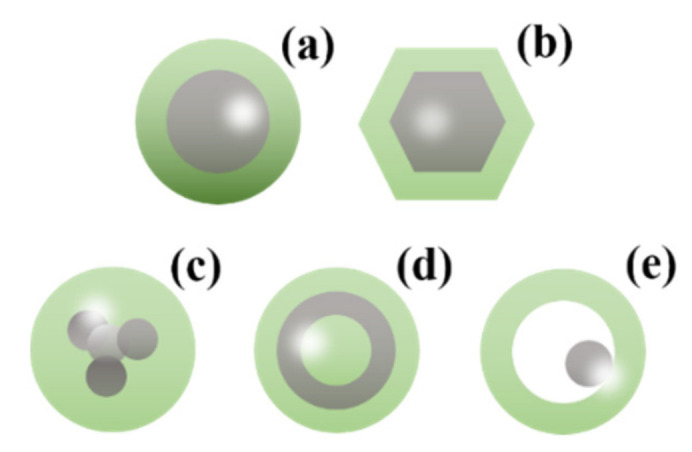
Different core-shell structures bimetallic nanocrystals: (**a**) spherical core-shell; (**b**) hexagonal core-shell; (**c**) multiple core with one shell; (**d**) multilayered core-shell; (**e**) yolk-shell [59].

In recent years, bimetallic nanocrystals with the core-shell structure have drawn widespread attention because of their improved performance over the combination of the two distinct metals in catalytic, energy and sensing applications. The core-shell structure (C@D) is an ordered assembly structure, which is composed of an inner core (metal C) completely coated with a shell (metal D) by chemical bonds or other forces [62]. When the precursors of the two metal ions have different reducing abilities, the precursor with the higher reduction potential could be preferentially reduced to the metal atom and form the nucleation center, and then the metal ions with the lower potential are reduced to metal atoms and deposited on the nucleation center, finally forming the core-shell structure (Figure 3). The most effective method to synthesize a core-shell structure is seed-mediated growth, in which metal C is often preferentially reduced and then acts as a heterogeneous nucleation site for metal D atoms to grow by chemical reduction or thermal decomposition methods [63,64,65,66]. The bimetals with multilayered nanostructures can be also generated by repeating the above process. After the efforts of many researchers, the nice thing is that the shell thickness of bimetal with core-shell structure could be precisely controlled at the atomic level [67,68], which is appealing for some special physicochemical properties, such as tuning optical properties, improvement in catalytic activity and stability, and augmenting of magnetic anisotropy. The core-shell structure has many morphologies due to the diverse morphologies of the seed (such as nanorod, nanowire, nanosphere, nanoplate, and polyhedra). In addition, the core-shell structure has many derived structures, such as multiple cores with one shell (Figure 3c), multilayered core-shell (onion structure/Figure 3d) and yolk-shell (Figure 3e).

The catalytic activities of the core-shell structured nanocrystals mainly depend on the composition of the shell metal, showing a catalytic effect similar to that of the single shell metal. Due to the poor electronegativity between the inner and outer metals, there will be electron transfer at the interface between the two distinct metals. Moreover, many researchers have paid attention to the dependence of atomic layer number on catalytic performance for bimetallic nanocrystals [69,70]. When the shell metal has only a single atomic layer, strain effect and electronic coupling effect are very significant, which could affect the catalytic properties including activity and selectivity of the shell metal. In this case, the system has the highest utilization efficiency of shell atoms, so that the material cost of surface atoms is the lowest. When the thickness of shell metal increases to multiple (2–6) atomic layers, strain and electronic coupling effect of core and shell atoms can still exist. Wang and co-workers studied the shell thickness-dependent catalytic activity of Pd@Pt_nL_ core−shell icosahedra, and result showed a volcano-shaped curve, and the optimal shell thickness is 2.7 nm [69]. When the thickness of the outer metal (metal D) is greater than six atomic layers (or more than 1.5 nm), some special properties of bimetal (strain and electronic coupling effect) may disappear, and thus the ability to modulate their catalytic properties is lost. However, other excellent properties about multilayer core–shell bimetallic nanocrystals are reflected, such as adjusting the optical properties, and improving the catalytic activity and selectivity of bimetallic catalyst. The above results, as well as other studies, fully proved the importance of accurate control of shell thickness in preparing a core-shell bimetal (C@D) for practical application.

### 2.3. Heterostructure

**Figure 4:**
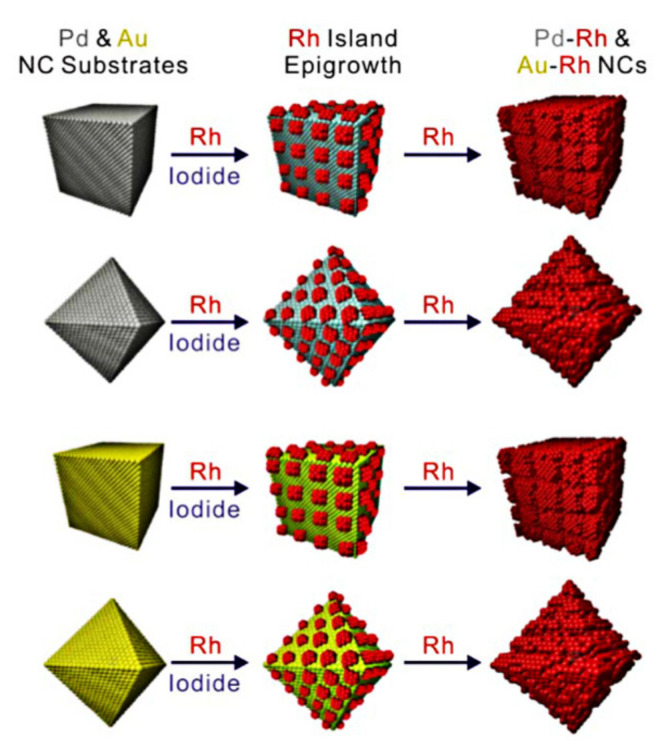
The synthetic diagram of controlling rhodium epigrowth on noble metal. Reproduced with permission from [71]. Copyright American Chemical Society, 2012.

When a metal atom is deposited on the surface of a substrate composed of another metal, there are three growth modes, namely, layer-layer growth mode, film-island growth mode, and island growth mode. When the D–D bond energy of metal D is greater than the C–D bond energy between metal C and metal D, or the specific surface free energy of D is greater than the specific surface free energy of C, D will choose the island growth mode because it can reduce the lattice stress between C and D and the total surface free energy. Simply put, when a metal preferentially generates nanocrystals with a certain morphology, and another metal atom adopts an “island growth mode” on the crystal, a multi-level structure will be formed (Figure 2d). For example, the Tsung research group synthesized PdRh heterostructure nanocrystals by Pd seeds [71]. Because the bond dissociation energy of Rh–Rh is greater than that of Rh–Pd, the Rh atoms generated by subsequent reduction will choose “island growth mode” on Pd nanocrystal surface (as shown in Figure 4).

The characteristics of heterostructure nanocrystals are similar to those of the mixture formed by mixing metal nanocrystals with corresponding sizes. The XRD pattern of the heterostructure bimetallic nanocrystals will have the same metal characteristic peaks as the two-metal mixture. On the one hand, the bimetallic nanocrystals with multi-level structure may retain the physical and chemical properties of the two distinct metals in the metal mixture; on the other hand, due to the interface interaction between multi-level structures, they will have some new properties different from the metal mixture in some systems [72,73].

### 2.4. Core-Frame and Other Derivative Structure

**Figure 5:**
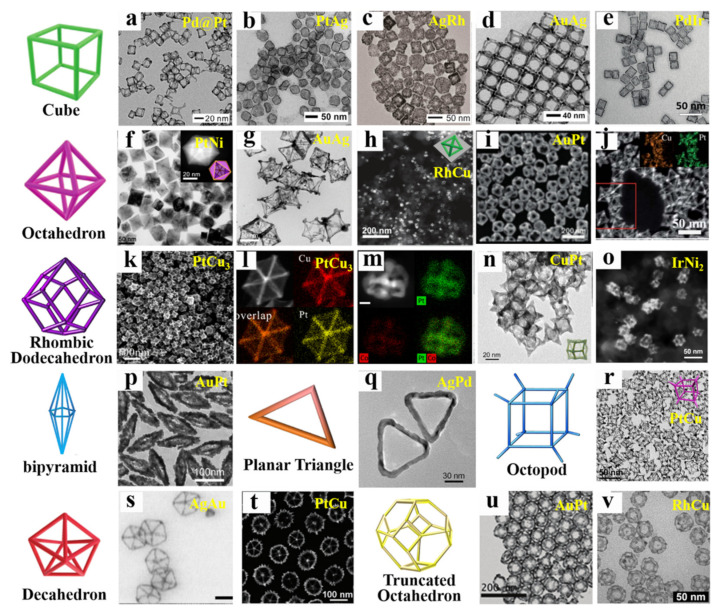
Summary of bimetallic nanoframe structures have been successfully prepared. Cube (**a**) Pd@Pt, Reproduced with permission from [81]. Copyright Wiley-VCH, 2016. (**b**) PtAg, Reproduced with permission from [94]. Copyright Royal Society of Chemistry, 2017., (**c**) AgRh Reproduced with permission from [95]. Copyright American Chemical Society, 2018. (**d**) AuAg, Reproduced with permission from [82]. Copyright American Chemical Society, 2018. (**e**) PdIr, Reproduced with permission from [96]. Copyright Wiley-VCH, 2019. Octahedron: (**f**) PtNi, Reproduced with permission from [97]. Copyright American Chemical Society, 2015. (**g**) AuAg, Reproduced with permission from [83]. Copyright Springer Nature, 2019. (**h**) RhCu, Reproduced with permission from [98]. Copyright Royal Society of Chemistry, 2015. (**i**) AuPt, Reproduced with permission from [99]. Copyright Wiley-VCH, 2019. (**j**) PtCu, Reproduced with permission from [86]. Copyright Royal Society of Chemistry, 2013. Rhombic Dodecahedron: (**k**) PtCu_3_, Reproduced with permission from [100]. Copyright American Chemical Society, 2014. (**l**) PdCu_3_ [100], (**m**) PtCo Reproduced with permission from [101]. Copyright American Chemical Society, 2020. (**n**) CuPt, Reproduced with permission from [102]. Copyright American Chemical Society, 2017. (**o**) IrNi_2_, Reproduced with permission from [90]. Copyright American Chemical Society, 2018. Bipyramid: (**p**) AuPt, Reproduced with permission from [85]. Copyright Wiley-VCH, 2019. Planar Triangle: (**q**) AgPd, Reproduced with permission from [103]. Copyright Wiley-VCH, 2015. (**r**) PtCu, Reproduced with permission from [88]. Copyright American Chemical Society, 2017. Decahedron: (**s**) AgAu, Reproduced with permission from [82]. Copyright American Chemical Society, 2011. (**t**) PtCu, Reproduced with permission from [87]. Copyright Wiley-VCH, 2016. Truncated Octahedron: (**u**) AuPt, Reproduced with permission from [75]. Copyright Royal Society of Chemistry, 2019. (**v**) RhCu, Reproduced with permission from [104]. Copyright Wiley-VCH, 2016.

In addition to the three basic nanostructures mentioned above, there are many derivative nanostructures, such as core-frame [18,74,75], nanodimers [76], hollow nanoboxes [77] and nanocages [78,79], and dendritic structure [80]. As we all know, the specific surface area and number of active catalytic sites must be maximized for effective catalysis. Compared with other structures, bimetallic nanocrystals with core-frame structure possess the maximum surface area per volume (high atomic efficiency) due to the hollow nanostructures with openings in their shells, which exhibited excellent electrocatalytic activity in terms of the oxygen reduction reaction (ORR), oxygen evolution reaction (OER), hydrogen evolution reaction (HER), and methanol oxidation reaction (MOR). Several research groups, including Xia and Lee, have done a lot of works on preparing the bimetallic nanocrystals with core-frame structure. The synthetic routes of nanoframes mainly include presynthesized template-assisted and solvothermal route involving in situ formed template. The second metal is grown on the template, and then the template is removed by acid etching or electric displacement to obtain a bimetal frame structure. Metals commonly used as templates are noble metals (Pd [81], Ag [82,83,84] and Au [75,85]) and transition metal (Cu [86,87,88] and Ni [89,90]). Some synthesized frame structures developed up until now are shown in Figure 5. The frame structures, including nanoboxes and nanocages, are the highly open structures with large specific surface area and atomic utilization, which often exhibit high catalytic activity and tunable spectral properties. Therefore, the nanocrystals with the open frame structures have been employed in many practical applications in some advanced fields, such as drug-delivery [91], photothermal cancer therapy [92], bio-imaging, sensing [93], fuel cell [90] and many others. However, there are shortcomings and limitations that need further study, such as instability.

## 3. Controllable Synthesis of Bimetallic Nanocrystals

It has been proved that the natural properties of bimetallic nanocrystals are closely related to their composition, size and shape. Xia and co-workers prepared Pd, PdPt and PdAu bimetallic nanocrystals with the same morphology by a seeded growth method for catalytic oxidation of formic acid, and the result showed that Pd concave decahedra exhibited improved catalytic activity, which was attributed to the high-index facets and twin boundaries on the Pd surface [105]. Therefore, the controllable synthesis of bimetallic nanocrystals is of great significance for further optimizing their functionality and application performance. Generally speaking, the growth process of bimetallic nanocrystals can be divided into two stages: nucleation and growth. The two processes are often continuous and difficult to separate, and the thermodynamic and kinetic processes of these two stages are often difficult to control, so the controllable synthesis of bimetallic nanocrystals becomes very complex. In the whole synthesis process, reaction temperature, environmental pressure and reaction time will affect the morphology of the crystal. In addition, the type, concentration and reduction potential of reactants, reducing agent and the surfactant will also affect the reduction rate of reactants and the nucleation and growth process of nanocrystals [17,106]. Thanks to the efforts of many research groups, many synthesis strategies have been developed to obtain bimetallic nanocrystals with well-controlled properties. It is worth noting that different synthesis methods also may produce the products with similar structure owing to the similarity of basic mechanisms or reaction pathways. The complex nanostructures are generally synthesized by a combination of two or more different synthetic methods. This paper will focus on the typical and most used synthetic strategies, including co-reduction, seed-mediated growth, thermal decomposition, and galvanic replacement reaction.

### 3.1. Co-Reduction

**Figure 6:**
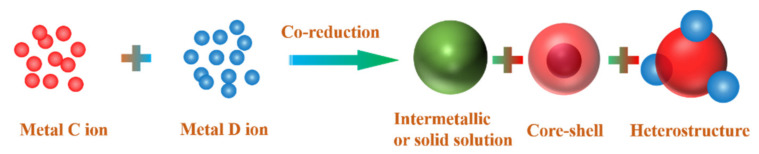
Schematic diagram of synthesis process by co-reduction method.

The co-reduction method entails that the precursors of two metal ions can be reduced simultaneously under certain reaction conditions, and then nucleate and grow into nanocrystals. The method is considered to be the most simple and straightforward strategy to prepare the bimetal with alloyed or intermetallic nanostructures [16] (Figure 6). However, core-shell and heterostructure also can be obtained by this method under certain condition. According to the study of LaMer [107], nucleation and growth should be divided into two distinct processes in order to obtain highly monodispersed nanocrystals. The nucleation and growth process of bimetallic nanoparticles mainly depends on the reduction rate of metal ions precursors, which is closely related to the reduction potential of metal ions. It is known that the higher reduction potential of metal ions, the easier the metal ions are to be reduced. The reduction potentials of the metal ions are shown in Table 2. Generally, metals with similar reduction potentials tend to form alloys. The two metals with large difference in reduction potential tend to form bimetallic with core-shell structure or heterostructure. In addition, the composition and shape of bimetals are related to the reactants (precursor, reducing agent and surfactant) and the reaction conditions (temperature, pressure and so on). According to the different reaction routes and conditions, the co-reduction method can be divided into solvothermal methods and oil-phase methods.

#### 3.1.1. Solvothermal Method

This method immerses the reactant in water or organic solvent, and then it reacts for a period of time in a closed container at a specific temperature, which can effectively control the structure and crystal phase of the nanomaterial. This solvothermal reaction has unique advantages and broad application prospects. Under solvothermal conditions, the entire system is in a state of high temperature and high pressure, the solvent is in a subcritical state, and the solubility is enhanced, so the activity of the reactants is greatly improved, and metastable phases that are difficult to obtain under normal pressure can be prepared. The solvothermal reaction parameters are easy to adjust and the nanocrystals with higher crystallinity and small size distribution can be effectively obtained. In addition, the growth mode of nanocrystals is also related to crystal face selection reagents. Therefore, in the solvothermal process, surfactant can be added to control the morphology of nanocrystals. Different crystal planes have different atomic arrangements, and organic reagents and inorganic ions have different adsorption capacities on different crystal planes, so nanocrystals can be controlled to grow in a specific direction.

**Figure 7:**
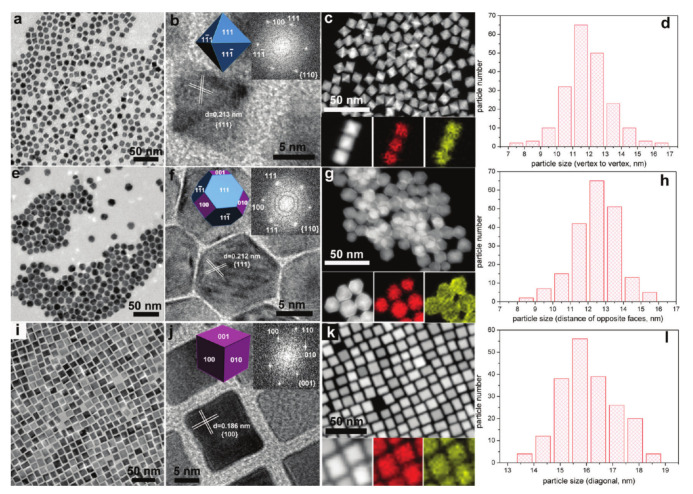
TEM (**a**), HRTEM (**b**) and HAADF-STEM (**c**) images of PtNi_2_ octahedrons and (**d**) corresponding size distribution, TEM (**e**,**i**), HRTEM (**f**,**j**), HAADF-STEM (**g**,**k**) images of PtNi_2_ truncated octahedrons and nanocubes, and (**h**,**l**) corresponding size distribution, respectively. Reproduced with permission from [108]. Copyright American Chemical Society, 2012.

The general idea for the synthesis of water-soluble metal nanocrystals is to select appropriate metal precursors (metal inorganic salt), hydrophilic solvents, surface ligands (e.g., PVP or CTAB) and morphology control agents (small inorganic molecule). For example, Wu and co-workers successfully prepared Pt–Ni alloy (PtNi_2_) nanocrystals with uniform size and morphology by solvothermal method [108]. The truncated octahedral nanocrystals can be controlled by the ratio of aniline to benzoic acid, and PdNi_2_ nanocrystals could be obtained by introducing the KBr in the system (Figure 7). Other bimetallic nanocrystals (e.g., PdNi, PtCu, PdCu) were also obtained by the above method [108].

#### 3.1.2. Oil Phase Method

**Figure 8:**
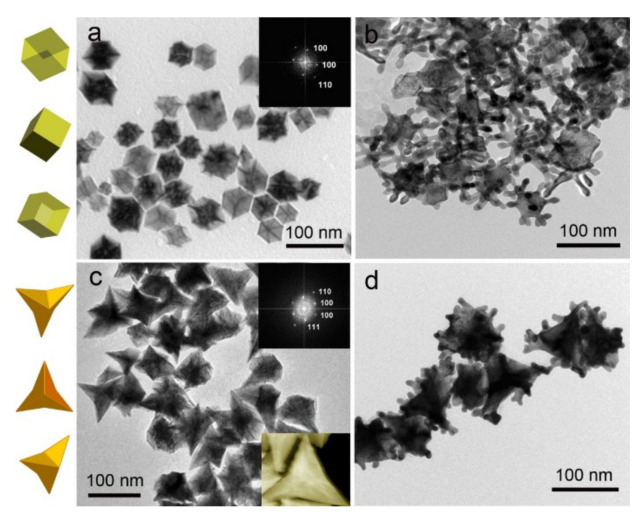
The structure evolution of Pt_x_Ni_1−x_ architecture: (**a**) PtNi_2_ (cubic), (**b**) PtNi_2_@Pt, (**c**) Pt_2_Ni (concave-tetrahedral, (**d**) Pt_2_Ni@Pt. Reproduced with permission from [109]. Copyright Royal Society of Chemistry, 2014.

When a high-boiling point solvent is used as a reaction medium, the metal precursor can nucleate explosively above the decomposition temperature, so the nucleation and growth stages can be better separated, which facilitates the preparation of nanocrystals with uniform size and good dispersion. Before the temperature rises, the reaction system can be evacuated; during the temperature rise and stability stage, an inert gas can be used to fill the system in order to exclude oxygen. Therefore, for the oxygen-sensitive preparation reaction process, the oil phase high temperature method has advantages that the hydrothermal reaction does not have. The high boiling point solvents are mostly long alkyl chain surfactants, which have a strong ability to coat nanoparticles. Therefore, it is necessary to eliminate the ligands on the surface before being applied to the catalytic reaction to release the catalytic active sites. For example, our group prepared Pt_2_Ni alloy@Pt nanocrystals (Figure 8) by using H_2_PtCl_6_∙ 6H_2_O and NiCl_2_∙6H_2_O as precursors and oleylamin as solvents and reducing agents [109]. Structure evolution of Pt_2_Ni alloy@Pt architectures were investigated by varying the precursor ratio. When Pt/Ni ratios were varied (1:2, 1:1, 2:1 and 3:1), the morphology of the products are shown as in Figure 8a–d. The different shapes can be ascribed to the increasing Pt concentration and different reduction potentials of Ni^2+^/Ni and Pt^4+^/Pt. The ORR performance exhibited that the order of catalytic activity is PtNi_2_ < PtNi_2_@Pt < Pt_2_Ni < Pt_2_Ni@Pt.

### 3.2. Seed-Mediated Growth

**Figure 9:**
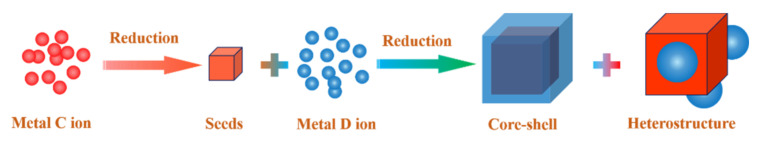
Schematic diagram of synthesis process by seed-mediated growth method.

**Figure 10:**
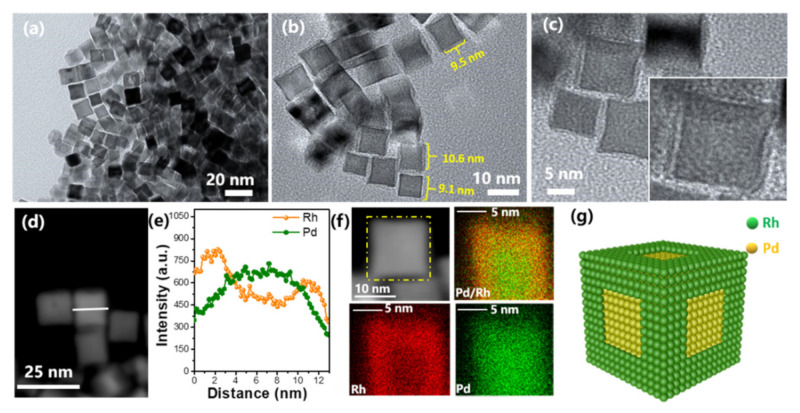
Schematic diagram of synthesis process by seed-mediated growth method. (**a**,**b**) TEM images and (**c**) HRTEM image of the PdRh bimetal SC. (**d**,**e**) High-resolution element line scan and (**f**) surface scan of the PdRh bimetal SC. (**g**) Structure model of PdRh SC. Reproduced with permission from [118]. Copyright American Chemical Society, 2020.

Seed-mediated growth is an effective synthesis strategy to generate the bimetallic nanocrystals with well-defined structures that are difficult to obtain by other methods [110,111,112,113]. For example, bimetallic nanocrystals with core-shell or heterostructure could be easily synthesized by this method. The conceptual diagram of these two structures obtained by the seed growth method is shown in Figure 9. In this process, one metal is firstly synthesized as a seed, and then the other metals are uniformly deposited on the seeds surface and form a shell layer, resulting in a bimetallic nanocrystal with a core-shell structure; and if the second metal is only deposited and grown in the special sites of seed crystals, the bimetallic nanocrystal with a heterogeneous structure will be obtained. The seed-mediated growth method was first put forward in 2001 by Murphy, who prepared Au nanorods by using Au nanoparticles as seeds [114]. Soon after that, Xia’s group prepared Ag nanowires by using Pt seeds [115]. In the next 10 years, the seed-mediated method was further expanded to prepare other bimetals (Pt@Pd [28], Au@Ag [116], Pd@Ag [117], and so on). The activation energy required for homogeneous nucleation is greater than that for growth. Therefore, when the solution contains pre-added seeds, the second metal ions will nucleate and grow heterogeneously on the seed surface after being reduced. This growth method has two growth modes: conformal epitaxial growth and non-conformal epitaxial growth. Different growth modes mainly depend on the reduction rate of metal ions, surface free energy, metal bond energy, and the choice of crystal plane stabilizing reagents. Yang and co-workers prepared Pt@Pd nanocrystals with different shapes (cubes, cuboctahedra, and octahedra) by using cubic Pt seeds [28]. They demonstrated whether the crystal lattice matches determined the growth mode of the crystal (isotropic growth or anisotropic growth). Lattice-matched Pd could produce conformal Pt@Pd core–shell nanocrystals, but Au grows anisotropically on the surface of Pt seeds due to distinctly different lattice parameters of Au and Pt. In addition, they found that the growth rates along different crystal directions could be altered by introducing NO_2_ into the reaction system and Pd (100) direction grows faster than that of Pd (111). This may be ascribed to the fact that Pd catalyst could dissociate NO_2_ to generate NO and atomic oxygen, and Pd (111) will adsorb dissociative oxygen and lead to partial passivation. After more than 10 years of hard work, this method has been widely used to prepare bimetallic nanocrystals with a core-shell structure. For example, our group synthesized Pd@Rh bimetallic nanocube by using Pd nanocube as seeds [118], and PdRh hollow nanoframes were obtained by etching Pd@Rh nanocube in HNO_3_ aqueous solution (as shown in Figure 10).

### 3.3. Thermal Decomposition

The principle of thermal decomposition is to heat organic metal compounds and decompose them to obtain metal nanoparticles. It is usually suitable for the preparation of metal nanoparticles with low reduction potential, and the method is a typical strategy for obtaining highly monodisperse nanocrystals. The thermal decomposition method was first proposed by the Thomas research group and used to prepare Ru-based bimetallic nanoparticles (RuPt, RuPd, RuSn and RuCu) [119]. Similar to the chemical reduction method, the composition, morphology and size of bimetallic nanoparticles prepared by thermal decomposition method are greatly affected by the proportion of metal precursors, the type of surfactant, solvent, reaction temperature and time.

Thermal decomposition has been demonstrated a simple and effective synthesis method to prepare various types of nanocrystals including mono- and bimetallic, and metal−oxide. It is known that the metal ion with relatively low reduction potential (such as Fe, Co, and Ni in Table 2) is difficult to reduce by chemical reduction method [120,121,122]. However, thermal decomposition can easily solve this problem. In the process of preparing bimetallic nanocrystals by the thermal decomposition method, some commonly used metal precursors mainly include the carbonyls (M(CO)_x_) and acetylacetonates (M(acac)_x_) [123,124], which could be easy to decompose under moderate conditions.

If the metal precursor is composed of two different metal complexes and their decomposition temperatures are quite different, the required alloy structure may not be obtained by direct pyrolysis. For example, Bönnemann and co-workers synthesized a series of Fe-Co nanocrystals by the simultaneous thermal decomposition of Fe(CO)_5_ and Co_2_(CO)_8_ in tetrahydronaphthalene [125]. Due to the different decomposition temperature between Fe(CO)_5_ (200 °C) and Co_2_(CO)_8_ (150 °C), their reaction rates were obviously different under the same condition. When the reaction temperature was 180–200 °C, monometallic nanocrystals (Co and Fe) were obtained as a result of the faster decomposition rate of Co_2_(CO)_8_ than Fe(CO)_5_. However, Fe-Co alloy could be obtained in the presence of aluminum trialkyl as catalyst at 150 °C. Therefore, when the pyrolysis temperature of the two metal precursors is quite different, it is necessary to introduce the corresponding catalyst to achieve the preparation of bimetallic alloy nanocrystals.

Although the large difference between the pyrolysis temperatures of the two metal complexes is not conducive to the preparation of bimetallic NPs, the existence of this difference also makes it possible to prepare core-shell bimetallic NPs by thermal decomposition method. The core-shell bimetallic NPs can be obtained by a thermal decomposition method. Firstly, the reaction is carried out at the lower decomposition temperature (lower temperature) of the more unstable metal complex to obtain the “core” layer of single metal nanoparticles, while the other more stable metal complex remains unchanged in the reaction system. Then, the reaction temperature is increased or the reaction time is prolonged to make the more stable metal complex decompose. The metal atoms are deposited on the surface of the single metal nanoparticles formed to obtain core-shell bimetallic nanoparticles.

### 3.4. Galvanic Replacement Reaction

**Figure 11:**
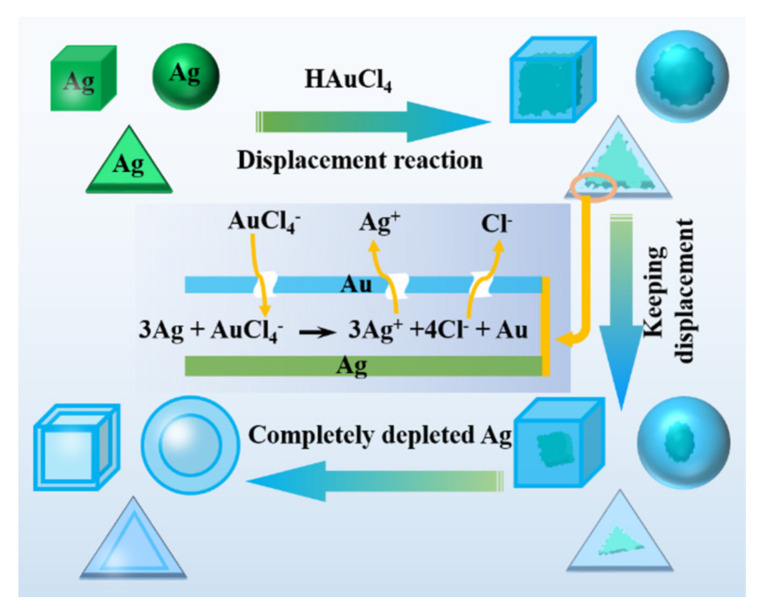
Schematic diagram of the experimental procedure that prepared hollow Au nanocrystals by using Ag nanocrystals with various morphologies as templates. Reproduced with permission from [126]. Copyright American Chemical Society, 2002.

As a good way to produce bimetals with special structure, the galvanic replacement reaction is an electrochemical process in which one metal is replaced by another metal ion due to their different reduction potential in the reaction system. In essence, the reaction is also an oxidation–reduction process. Metal (C) with high potential can be easily oxidized. Metal ions (D^+x^) with low reduction potential are difficult to reduce by chemical reduction method, but can be reduced by metal C. Therefore, the method is usually suitable for preparing the inactive metal nanocrystals using an active metal as the template. Xia’s research group have prepared a lot of bimetals including one active metal and one inactive metal by the galvanic replacement reaction [126,127]. They used Ag as a template, and a salt solution of Au, Pt, and Pd as precursors, to prepare hollow nanoparticles of Au, Pt, and Pd by a galvanic replacement reaction at a certain temperature [126], and the schematic diagram of reaction process are displayed in Figure 11. The nanostructure of Ag used as a template will affect the morphology and wall thickness of Au nanocrystals. The metal with strong reducibility acts as a template to some extent. In addition to Ag, many other metals can also be used as templates, such as Pd [128], Ni [129,130], Co [129,131,132], Pb [129], Cu [133], Se [134] and Te [135]; For example, Mikhlin and co-workers [126] firstly made Pd (a metal with relatively high reducibility) nanoparticles with a specific configuration, and then placed it in a solution of Au^3+^ with a higher oxidation potential. Au^3+^ will be reduced to zero valence (Au^0^) and then deposited on the Pd NPs surface, thereby forming Pd-Au nanocrystals with the core-shell structure.

### 3.5. Other Methods

Thanks to the nanoengineering technique, the morphology and microstructure of the nanoparticles can be precisely controlled. In addition to the four methods we mentioned above, there are some other synthetic protocols to prepare bimetals with well-defined shapes, such as hard or soft template methods, combustion synthesis, hydrothermal treatment, the combination of the two approaches and so on. These methods have their own advantages and disadvantages in preparing bimetallic nanocrystals with different special structures. The advantages and disadvantages of the four most common methods for preparing bimetallic nanocrystals are shown in Table 3.

## 4. Applications of Bimetallic Nanocrystals

Bimetallic nanocrystals have been widely used in many important fields from science to technology [16,17,136,137,138]. In comparison to single metal nanocrystals, bimetallic nanocrystals possess many unique properties due to the adjustable composition, controllable morphology and variable electronic structure, so they also have many practical and potential applications including catalysis, sensing, biodetection, biomedicine, and so on. For biomedicine, because biological systems are complex, the use of biomaterials are also strict, which requires not only biological activity and biocompatibility, but also requires that the harmful effects after use can be easily eliminated. Considering these factors, single metal nanocrystals usually cannot meet the requirements, while multi-functional bimetallic nanocrystals are more suitable for biomaterial applications. In addition, Pt catalyst has been proved to have excellent catalytic performance for ORR and methanol oxidation reaction (MOR). However, the reserves of Pt and Ir in earth are limited, and the catalytic performance of pure Pt catalyst is not perfect either. Therefore, the introduction of the second metal to form bimetallic nanocrystals is a good solution for improving some important chemical reactions (MOR, ORR and OER). With the development of synthetic bimetallic nanoparticles technology, the micro morphology, carrier properties and composition of bimetallic nanoparticles can be adjusted according to the requirements of different chemical reactions for catalyst activity to obtain the best catalytic activity. In the chemical reactions with important industrial application, a slight improvement in activity, selectivity and stability can bring considerable economic savings. In this section, we have summarized several important applications of bimetallic nanocrystals, such as electrocatalysis, heterogeneous catalysis, sensing and biodetection application, and the composition-dependent and shape-dependent properties of bimetallic nanocrystals in these applications are also discussed.

### 4.1. Catalytic Applications

Bimetallic nanocatalysts have been widely used in solution electrocatalytic reactions, such as methanol electrooxidation, hydrogen electrooxidation, and oxygen reduction reaction and so on. At the same time, they are often used to catalyze heterogeneous reactions, such as CO oxidation, NO_x_ reduction, and petroleum reforming and so on. The applications of bimetallic nanocrystals in heterogeneous catalytic reactions such as anode and cathode of fuel cells are briefly introduced.

#### Electrocatalysis

1. Oxygen Reduction Reaction (ORR)

The polymer electrolyte membrane fuel cell (PEMFC) is expected to become one of the alternative energy sources for fossil fuels. However, its large-scale application is mainly limited by the kinetics of cathodic reaction, i.e., ORR, including the existence of cathodic overpotential and the loss of catalyst in the process of cathodic reaction [139]. The catalyst should have enough activity to activate O_2_ and enough inertia to prevent itself from being oxidized. Compared with Fe, Co, Ni, Ag, Au and other metals, Pt has higher active sites and moderate adsorption and desorption energies for OH, O and other cathode intermediate products, so it is an excellent catalyst for the ORR reaction [140,141,142,143]. Because Pt is a noble metal, and the loss of Pt in the catalytic process is serious, looking for a Pt based alloy with high activity and good stability to replace pure Pt has become a research hotspot in the development of fuel cells. For example, when Pt is alloyed with 3d transition metals, it can not only reduce the cost of catalyst, but change the d-band electronic structure of surface Pt atom by electron transfer and improve the catalytic performance [144].

**Figure 12:**
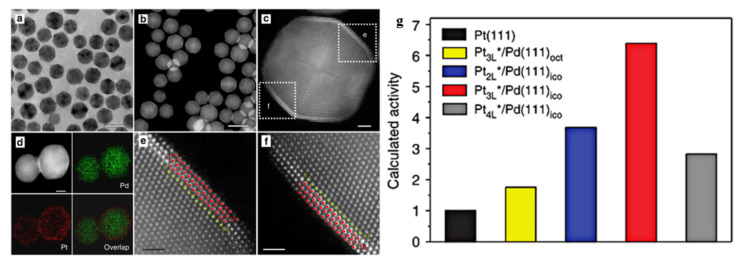
(**a**) TEM and (**b**) HAADF-STEM images. Scale bar, 20 nm. (**c**) Atomic-resolution HAADF-STEM image Scale bar, 2 nm. (**d**) HAADF-STEM image of Pd@Pt_2.7L_ icosahedra and the corresponding mapping of Pd and Pt. Scale bar, 5 nm. (**e**,**f**) Atomic-resolution HAADF-STEM images corresponding to (**c**) revealing the detailed arrangements of Pd (green dots) and Pt atoms (red dots). Scale bar, 1 nm. (**g**) The catalytic activity of Pt_nL_/Pd(111)_ico_ to ORRs according to the result of DFT calculations. Reproduced with permission from [150]. Copyright Springer Nature, 2015.

For the ORR reaction, the catalytic activity of Pt based alloy is not only related to the composition, but also to the exposed crystal surface. The atoms on different crystal planes have different coordination conditions. The difference of coordination number leads to different electronic filling degree of metal d-band, leading to a different adsorption capacity. For instance, Stamenkovic and co-workers investigated the relationship of ORR activity and different Pt_3_Ni single-crystal surfaces by using the ex situ and in situ surface-sensitive probes and DFT calculations, and found that the ORR activity on Pt_3_Ni (111) facet was 10-fold higher than that of Pt (111) facet, and 90-fold higher than that of commercial Pt/C catalyst at that time [139]. The significantly improved performance of the Pt_3_Ni (111) facet was proved to be due to the special electronic structure and weak interaction of Pt atoms on the surface, which increased the number of active sites. After that some PtNi nanostructures were prepared, such as PtNi/Pt-skins [140], Pt_3_Ni octahedral [145], Pt_3_Ni truncated octahedral [146], Pt_3_Ni icosahedra [147], Pt_x_Ni_1−x_ octahedral [148], and the Pt_3_Ni nanoframe [149,150]. According to the Nørskov’s study [141], the reaction activity of ORR depends largely on the energy change during the formation of hydrogen peroxide and the kinetic process during the formation of water. When the ability of adsorbing O_2_ and O is strong, the ability of desorbing OH is weak. The desorption of -OH is the decisive step of ORR. For instance, Xia and co-workers [69] prepared Pd@Pt_nL_ core shell catalysts with different platinum overlayers (n = 0.7–4.3) by using Pd icosahedra as seeds (Figure 12), and specific activities and mass activities of Pd@Pt_2.7L_ catalyst to ORR were improved by eight and seven times, respectively, in comparison to a commercial catalyst. Moreover, Density functional theory (DFT) calculations demonstrated that the improvement could be ascribed to the weakened binding of hydroxyl to Pt surface growing on Pd (Figure 12g). Chuang [56] prepared PtFe nanocatalysts for electrochemical ORR, and found that ordered fct-PtFe/C catalyst coated with an N-doped carbon exhibited 11-fold mass activity and 10-fold specific activity compared with the commercial Pt/C, and exhibited excellent long-term stability.

**Figure 13:**
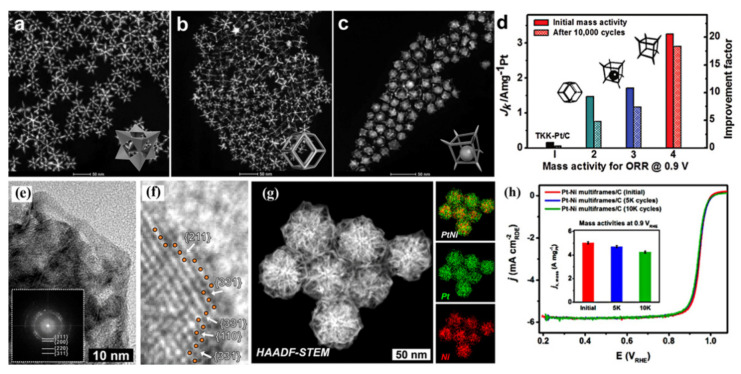
HAADF-STEM images of various PtCu nanoframes. (**a**) concave octopod, (**b**) rhombic dodecahedral, (**c**) PtPdCu yolk-shell, (**d**) comparison of ORR mass activities at 0.9 V RHE of various catalysts before and after cycling tests for 10,000 cycles in 0.1 m HClO_4_. Reproduced with permission from [151]. Copyright Wiley-VCH, 2016. (**e**) HRTEM image of the Pt−Ni multiframes. (**f**) Enlarged HRTEM image. (**g**) HAADF-STEM image and the corresponding elemental mapping. (**h**) ORR polarization curves and mass activities (inset) of the Pt−Ni multiframes/C catalyst. Reproduced with permission from [152]. Copyright American Chemical Society, 2018.

Pt-based catalysts with nanoframes exhibited excellent ORR activity due to the high atomic efficiency. Luo [151] reported that PtCu concave octopod nanoframes, rhombic dodecahedral (RD) nanoframes, and Pt–Pd–Cu yolk-shell nanoframes (Figure 13a–c) were prepared by altering the type of precursor, halide ion sources and adjusting the reduction order of the precursor. And the octopod nanoframe architectures showed high mass activity of 2.95 A mgPt^−1^, which was a 47-fold enhancement in comparison to the commercial TKK-Pt/C catalyst (Figure 13d). The significant improvement can be ascribed to the 3D porous structure, high-index facets, and a large number of active sites exposed on grain boundary edges. As shown in Figure 13e–h, Lee prepared the dendrite-embedded Pt−Ni multiframes by a facile one-pot method, and ORR performance demonstrated that dendrite-embedded Pt−Ni multiframes had high mass activity and catalytic stability, which can be ascribed to the active surface with high-indexed facets, porous hierarchical structure, and the synergy between the inner dendrite core and highly fused multiframes on the surface [152].

2. Methanol Oxidation Reaction (MOR)

A methanol fuel cell has high energy density and proton exchange membrane has high conductivity. Therefore, methanol fuel cell is considered as a promising cell with potential application, and is increasingly favored by chemical and material researchers. Transition metals, particularly Pt-based metals, are commonly used as catalysts of anode and cathode reactions in fuel cells. At present, the electrocatalytic methanol oxidation (MOR) is often used as a probe reaction to evaluate the comprehensive performance of metal catalysts. CO is the intermediate product of methanol oxidation, which easily causes catalyst poisoning and the loss of catalytic activity. Pt–Ru alloy is proved to be an efficient catalyst for methanol fuel cell because Ru can promote the formation of Ru-OH at lower potential, and Pt can regain the adsorption sites and promote the oxidation process of methanol. According to the Watanabe–Motoo (W–M) mechanism, the OH^-^ is adsorbed at the Ru site (Ru-OH), and the intermediate product of CO_ads_ formed at the Pt site. Ru can oxidize the formed CO_ads_ and avoid Pt active site poisoning. However, Tong and co-workers proposed another mechanism by their experimental results [153], in which the MOR occurs at the Pt–Ru boundary site, not a single metal site (Pt or Ru). In addition, the activation region of a PtRu catalyst is usually performed before the MOR [154].

**Figure 14:**
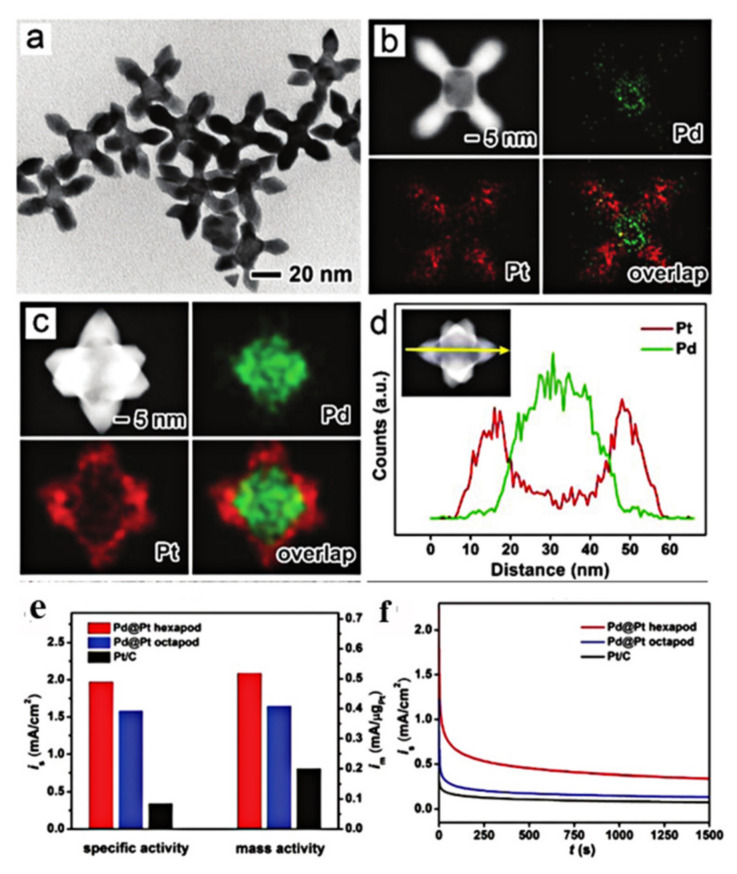
(**a**) TEM, (**b**) HAADF-STEM-EDX mapping images of the Pd@Pt octapod. (**c**) HAADF-STEM image and the corresponding mapping image, (**d**) EDX line-scan profile image of the Pd@Pt hexapods. (**e**) The comparison of specific and mass activity of the different catalysts (**f**) Current–time curves for methanol electro-oxidation of these three catalysts. Reproduced with permission from [156]. Copyright Royal Society of Chemistry, 2017.

PtPd and PtRu catalysts with different shapes and structure have been proved to be effective catalysts for MOR, because Pd and Ru can change the electronic structure of Pt [15,39,155,156]. In addition, PdPt nanocrystals also exhibited facet and composition-dependent catalytic activity for MOR. For instance, the PtPd nanocubes (exposed (100) facet) exhibited a higher activity than PtPd nanotetrahedrons (exposed (111) facet), whereas the (111) facet exhibited a better durability than (100) [26]. As shown in Figure 14, Yang and co-workers [156] prepared two kinds of Pd@Pt core–shell dendritic nanostructures (hexapod and octapod) by the seeded growth method for MOR. The result showed that hexapods possessed the highest specific and mass activity, and the best catalytic stability compared with octapod and the commercial Pt/C catalyst. In addition, two kinds of Pd@Pt core–shell dendritic nanostructures displayed a significantly improved catalytic performance in comparison to the commercial Pt/C, which was supposed to be due to the dendritic nanostructure and the synergy between Pd and Pt. Our group synthesized PtRh alloy 3D porous nanostructure with tunable atomic ratio (Pt and Rh) by a modified template-free self-assembly approach [157]. Three-dimensional porous nanostructure PtRh with a moderate Rh content (~15 wt%) exhibited high electrochemical activity and good anti poisoning performance for MOR compared with the commercial Pt/C and PtRh NPs. The unalloyed Rh atoms would result in a self-adjusted dissolution capability of PtRh catalysts by the dissolution of Rh atoms. In addition, the formation of Rh oxide in the initial 50 cycles played a significant role in enhancing the electrocatalytic activity for methanol oxidation. On the other hand, the activity of the catalyst depended not only on the exposed crystal surface, but also on the morphology of the particles. Pt–Pd nanodendrites [158], Pt–Pd nanocages [159], and Pt–Pd hollow core-shell structure were reported to be the effective catalysts for MOR. In order to save the usage of noble metals and improve the stability and durability, tertiary metals catalysts were also developed such as PtPdTe nanowires [160], PtPdFe nanowires [161], PtPdNi nanoarchitectures [162], and PtRuM (M = Fe, Ni, Co) [163]. Therefore, the synthesis of tertiary Pt-based nanocrystals should be a promising scheme to design efficient catalysts for MOR.

3. Oxygen Evolution Reaction (OER)

**Figure 15:**
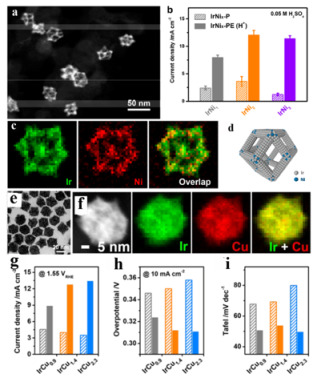
(**a**–**d**) Surface structural evolution of the IrNi*_x_*-P catalysts and Current densities of IrNi*_x_*-P. Reproduced with permission from [90]. Copyright American Chemical Society, 2018. (**e**–**i**) TEM image and STEM-EDX mappings of P-IrCu_1.4_, and polarization curves of S-IrCu*_x_* nanocrystals and P-IrCu*_x_* nanocrystals. Reproduced with permission from [172]. Copyright American Chemical Society, 2018.

Energy storage is a particularly important field to improve the utilization of renewable energy because it is difficult to use renewable energy anytime and anywhere [164]. Water decomposition has attracted a large number of researchers’ interest [137,165,166], which can convert electrical energy into chemical energy. However, water electrolysis is a demanding reaction due to the large overpotential at the OER [167]. In addition, the activity and stability of some catalysts to OER (Ru*_x_*Ir_1−_*_x_* alloys [167], SrRuO_3_ [168]) demonstrated an inverse relationship. Although Ir and Ru were proved to be the best catalysts for ORR, the cost is high due to the fact that they are rare metals. In order to obtain an excellent catalyst, the activity, stability and cost of catalysts should be balanced. Therefore, Ir-based bimetallic nanocatalysts with transition metals (Ti [169], Ni [90,170], Cu [171,172]) will be a good strategy to balance these factors. For examples, Huang et al. [90] constructed monodispersed IrNi*_x_* nanocrystals with distinct composition-segregated features (Figure 15a,c) and investigated their structural evolution in various OER environments. They found that Ni of IrNi*_x_* nanocrystals could migrate in various OER environments, and an Ir-skin framework was generated in an acidic electrolyte, while a Ni-rich surface layer was observed in an alkaline electrolyte. The Ni migration played a significant role on enhancing the catalytic activity for MOR. Moreover, they synthesized solid Ir-Cu nanocrystals by a facile chemical dealloying strategy and then porous Ir-Cu nanocrystals were obtained through etching with nitric acid. The porous Ir-Cu nanocrystals were highly active and stable toward OER in acidic conditions due to the increased active surface area and plenty of defect. Our group prepared IrNi alloyed nanocrystals by oil phase method for OER. The result showed that IrNi alloyed nanocrystals could enhance OER activity of flower-shaped NiCo_2_O_4_ electrode material [173]. IrNi/NiCo_2_O_4_ exhibited a relatively lower overpotential and a higher current density, which was ascribed to the synergic effect of the IrNi and NiCo_2_O_4_ and high catalytic activity of IrNi alloy.

### 4.2. Heterogeneous Catalysis

Heterogeneous catalytic reactions involve many physical and chemical transformations including the diffusion of reactant molecules in the pore, adsorption on the surface, reaction on the surface of the solid catalyst, desorption and diffusion of product molecules. For the catalyst, the adsorption center is often the catalytic active center. The relationship between catalytic performance (i.e., activity, selectivity, and durability) and catalyst properties, composition, size, and shape (more precisely, facet type) of the catalyst has been studied intensively for many years. With the development of synthesis technology of bimetallic NPs, the micro morphology, carrier properties and composition of bimetal can be adjusted as the requirements of different chemical reactions for catalyst activity to obtain the best catalyst. It lies in the subtle change of catalytic activity and selectivity in chemical reactions with important industrial applications. Therefore, as a design platform of high activity catalysts, bimetallic nanoparticles have great potential in the field of catalysis. Recently, a variety of bimetallic catalysts have been designed and applied to different types of chemical reaction, such as CO oxidation [174], hydrogenation/dehydrogenation [175,176,177], coupling reactions [178,179] and H_2_ evolution reaction (HER) [180,181,182], and showed superior catalytic activity. The composition, size, and morphology of a bimetallic catalyst has a great effect on its catalytic properties. In order to better understand the relationship between the structure and properties of bimetallic catalysts, two important types of reaction including H_2_ evolution reaction and carbon carbon coupling reaction will be discussed in detail.

Catalytic or photocatalytic hydrogen production is an effective means of energy regeneration and storage today. Generally speaking, hydrogen storage materials are mainly small molecules, such as sodium borohydride, hydrazine, lower alcohols and borohydrides, hydrides (LiH, MgH_2_, AlH_3_) and so on. It has been reported that the common hydrogen production catalysts are Ni-based catalysts (PtNi [181,183], RhNi [182], IrNi [184], PdNi [180,185], FeNi [186] and CoNi [187]), especially Rh-Ni and Pt-Ni alloy catalysts, which have the best activity and selectivity. Moreover, the composition and morphology of the alloy catalyst have a great influence on the performance of the catalyst. For example, Mao and co-workers [180] prepared PdNi hollow alloy with different stoichiometry by a galvanic replacement method for photocatalytic hydrogen evolution, and the order of the hydrogen generation rate is Pd_1_Ni_1_ > Pd_2_Ni_1_ > Pd_1_Ni_2_ > Pd. The enhanced performance can be ascribed to the stronger interaction between Pd_1_Ni_1_ hollow NPs and CdS than other alloys. In addition, recently the development of multi-element alloys and non-noble metal alloys have been proven to be a good way to solve the high cost and stability problems of noble metal catalysts [188,189]. Wang and co-workers investigated the catalytic performance of bimetals (PtCo and PtNi) and ternary alloy (PtNiCo) catalysts for hydrogen production, and the result showed that the PtNiCo catalyst exhibited excellent HER performance, superior to PtNi, PtCo and commercial Pt/C catalysts [188].

The carbon–carbon coupling reaction is an important kind of carbon increasing reaction, including Sonogashira-, Ullmann-, Suzuki coupling, etc., which plays a significant role in medicine, biochemistry and other industries. The research demonstrated that Pd based nanoparticles exhibited high catalytic activity for the carbon–carbon coupling reaction, and the component really played the catalytic role was zero valent Pd [190,191,192]. However, these coupling reactions are only carried out on the surface of nanoparticles, and most of the remaining Pd is wasted. Therefore, how to improve the utilization rate of Pd and reduce the amount of Pd used in this process is a challenging problem. The introduction of iron series elements (Fe, Co, Ni) into a Pd system can not only reduce the cost of the catalyst, but change the activity and selectivity of the catalyst by adjusting the electronic structure. The Hyeon group synthesized the Pd-Ni NPs with a Ni-rich core/Pd-rich shell structure by thermal deposition method, and the Pd/Ni NPs displayed higher catalytic activity and better recycling performance than pure Pd NPs. PdCo alloyed NPs displayed high catalytic activity to the Sonogashira coupling reaction, and the addition of Co could not only make the catalyst easier to recycle but be reused several times without significant loss of activity. In addition to increasing the composition, the preparation of PdM bimetal with special structure can also improve the catalytic performance. For example, Xia and coworkers [193] prepared the core–shell-like catalyst Ni-Pd/CB for Suzuki–Miyaura coupling reaction and the catalytic performances was enhanced significantly under mild aerobic conditions without using toxic solvents. In addition, the Ni core not only make the catalyst have magnetically separable ability, but also greatly improves the utility of the Pd element.

### 4.3. Energy Conversion Applications

CO_2_ is one of the most widely existing carbon sources in nature, but it is also the lowest energy form of carbon. Absorption and capture of CO_2_ and catalytic reduction of CO_2_ to high value-added compounds are also the means of conversion and utilization of CO_2_. The most mature industrial application of fixing CO_2_ by chemical method is to produce urea, ammonium bicarbonate and other fertilizers. However, the research on conversion of CO_2_ to other high value-added carbon containing small molecular compounds, such as hydrogenation to alkanes, olefins and other fuels, or to medium acid, ethanol and other chemical products, has not been extended to large-scale applications. The application of this process mainly depends on the development of high catalytic performance and low-cost catalysts. The preparation of CO, HCOOH, CH_3_OH, CH_4_, C_2_H_6_ and other organic hydrocarbon small molecular compounds by the electrochemical catalysis of CO_2_ have attracted the attention of researchers as a promising method for large-scale energy storage (power storage) of solar energy and wind energy due to its relatively mild conditions, strong controllability and high yield per unit area (compared with a direct photocatalytic method). At present, the catalytic conversion methods of CO_2_ are widely studied, including photocatalytic reduction, chemical catalytic reduction and electrochemical catalytic reduction.

Based on the continuous understanding of the properties of a single metal material, following Kuhl’s research idea of adjusting the CO binding energy of alloy materials to improve the catalytic activity and product selectivity, a large number of metal alloy materials have been used to catalyze the reduction of carbon dioxide in the last five years. The combinations of alloy materials mainly include noble metal noble metal, noble metal non noble metal and non-noble metal non-noble metal, which are controlled by material composition. Christopher and co-workers [194] prepared Pd_y_Au_(1-y)_ alloy (y = 0, 0.25, 0.45, 0.8) thin film and investigated their performance for the electrocatalytic reduction of CO_2_. And the result showed that all AuPd alloys had higher activity and better selectivity for formate than single Au or Pd. The improved performance of AuPd alloy was attributed to yield new electrocatalytic properties due to synergistic effect of gold and palladium.

Compared with the alloy materials with noble metal components, the bimetallic materials formed by two kinds of non-noble metal can also obtain high catalytic activity and selectivity if the size, shapes and compositions could be reasonably designed. For example, Abdesslem [195] reported that CuIn alloy were obtained by reducing CuInO_2_ and used to electrocatalytic reduction of CO_2_. The performance results showed that CuIn alloy catalyst had high catalytic activity and selectivity for CO_2_ reduction, which was ascribed to the generation of active sites that convert CO_2_ to CO. Noble metal Pd is a good catalyst for CO_2_ reduction, but it is easily poisoned by CO. The synthesis of Pd-based alloy catalysts is a good solution to overcome the problem. For example, Toshihiro [196] synthesized PdCu bimetallic catalysts and studied the influence of the catalyst composition on the CO_2_ reduction activity and the selectivity. The result showed that high Pd/Cu ratio led to production of formate rather than hydrogen, and PdCu exhibited excellent stability for CO_2_ reduction with a small overpotential due to the electrical interaction between Pd and Cu [196].

### 4.4. Sensing Applications

A sensor is a detection device, which can selectively recognize the target information and transform the information into electrical signal or other measurable signal according to certain rules. Under normal conditions, bimetallic NPs can be designed and obtained to express their unique properties of each metal. Therefore, a lot of effort has been devoted to preparing and developing bimetallic nanocrystals with well-defined structure (such as facets, atomic order and spatial arrangement). Thanks to the efforts of scientific researchers, many groups have already synthesized a large number of different bimetallic nanocrystals to meet people’s different needs. It is a fact that bimetallic nanocrystals have been widely used in many important fields because of their excellent properties, such as catalytic properties, optical properties, localized surface plasmon resonance (LSPR), photocatalytic properties and magnetic properties. etc. More importantly, the modification and doping of bimetallic nanocrystals especially noble metals (Pd, Pt, Au, Rh, and Ag) have been proved to be among the most effective ways to reduce the operating temperature of sensors, enhance the response of target gases and improve the selectivity of sensors.

#### 4.4.1. Metal Oxide Semiconducting (MOS) Sensors

Semiconducting gas sensors are composed of sensors with semiconducting metal oxide materials as the main materials like SnO_2_, ZnO, WO_3_. etc. After nearly 60 years of development, great progress has been made in the mechanism and materials, especially in the response value and selectivity. The modification and doping of bimetals have played a very significant role in improving their gas-sensing properties. For example, due to the complex detection environment, the diversity of gas molecules, and the characteristics of semiconducting gas-sensing materials, it cannot accurately distinguish different gases like acetone, ethanol, formaldehyde that have similar properties in the application, which is an urgent problem to overcome. The stability of semiconducting gas sensor is also a problem. Whether the sensors can detect the target gas stably is related to the production as well as life. However, owing to the development of science and technology, the modification and doping of bimetallic nanocrystals can solve this problem much better than ever before. Because different metals have different properties, we can design and custom-made the bimetallic nanocrystals structure and morphology of materials according to the requirements, so as to better solve the problem of selectivity.

**Figure 16:**
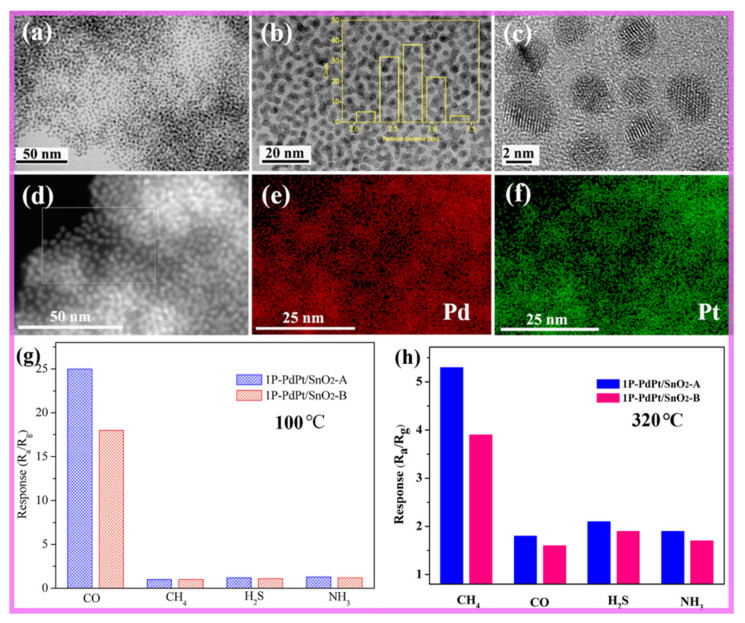
(**a**,**b**) TEM images and (**c**) HRTEM image of PdPt bimetal (inset (**b**) is the particle size distribution), (**d**) HAADF-STEM image, corresponding EDS mapping for (**e**) Pd, (**f**) Pt, selectivity of the obtained sensors on exposure to 1000 ppm methane and 50 ppm other gases at different working temperatures (**g**) 100 °C and (**h**) 320 °C. Reproduced with permission from [198]. Copyright American Chemical Society, 2019.

Bimetallic nanocrystals are expected to lower the operating temperature of the sensor and show some new properties. The research work of our group shows that gas sensors with dual selectivity can be obtained by decorating bimetallic nanocrystals on the surface of metal oxide semiconductor. For example, PdAu/SnO_2_ composite was prepared by decorating pre-synthesized PdAu nanocrystals on the surface of SnO_2_ NSs [197]. The response to acetone and formaldehyde were greatly improved by the decoration of bimetallic nanocrystal PdAu. At the same time, we found that the gas sensing material had double selectivity to different gases at different temperatures. At 250 °C, the response to 50 ppm acetone was 109, and the selectivity and stability to acetone were very good. When the temperature was 110 °C, the response to 50 ppm formaldehyde was 86, showing a good stability and sensitivity. Later, our group prepared bimetallic nanocrystals to detect coal mine gas. As shown in Figure 16a–d, PdPt NPs displayed good dispersion with a narrow size distribution from 2 to 3.5 nm (mean size = 2.7 nm). The sensing performance demonstrated that a PdPt bimetal-functionalized SnO_2_ nanosheets sensor could detect carbon monoxide at 100 °C and methane at 320 °C (Figure 16g,h) [198].

#### 4.4.2. Electrochemical Sensors

The mechanism of electrochemical sensors is based on ionic conductivity to the electrode and electrochemically active species, which can be oxidized or reduced on the electrode surface. Due to the economic benefits, simple operation, high selectivity and sensitivity, the research and application fields of electrochemical sensors continue to expand. Research in this field is still focused on new sensing strategies, especially on enhancing specificity, detection limit and response time. Nanomaterials are widely used in electrochemical sensors. One significant point is the research and application of bimetallic nanocrystals, which play a significant role in the development of electrochemical sensors. Due to electronic and synergistic effects, electrochemical sensors based on bimetals usually exhibit higher sensitivity and better selectivity than either of the two metals. For many years, scientists have been committed to the research and development of various electrochemical sensors, among which the electrochemical gas sensors based on bimetals have also been booming. Many electrochemical sensors are called electronic tongues, and research focuses on the development of liquid sensors and chemometric analysis of data.

**Figure 17:**
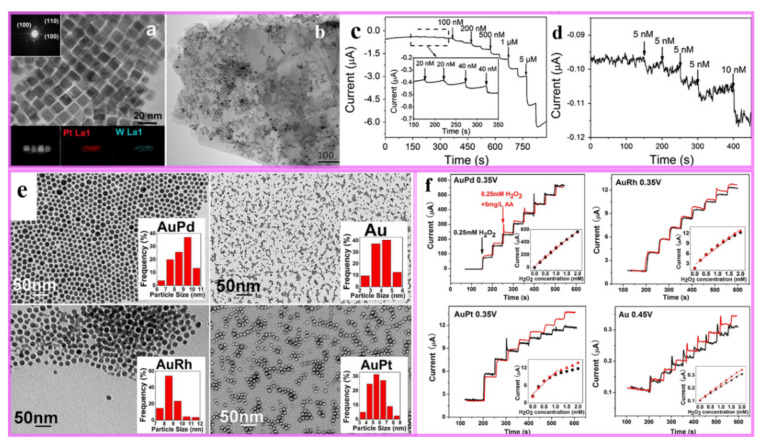
(**a**) TEM image of PtW nanocrystals, STEM image of random selection of several PtW nanocrystals and corresponding element maps; (**b**) TEM image of PtW/MoS_2_ nanocomposite; (**c**) Ultra-sensitive responses of PtW/MoS_2_ nanocomposite material towards successive addition of H_2_O_2_ at an applied potential of 0.25 V vs. SCE. Inset is the blow-up of low concentration region. (**d**) The detection limit of PtW/MoS_2_ nanocomposite material. Reproduced with permission from [199]. Copyright Elsevier, 2016. (**e**) TEM images of AuPd, AuRh, AuPt and Au nanocrystals; (**f**) Current–time responses to pure H_2_O_2_ (black line) and the mixture of H_2_O_2_ with AA (red line) for AuPd, AuRh, AuPt (0.35 V vs. SCE) and Au catalysts (0.45 V vs. SCE). Each injection of pure H_2_O_2_ was 0.25 mM, each injection of mixture containing 0.25 mM H_2_O_2_ and 5 mg/L AA. Reproduced with permission from [200]. Copyright Elsevier, 2015.

Owing to their simplicity, low energy consumption and the ease with which they can be miniaturized, electrochemical sensors are very suitable for the development of portable sensing devices. Up to now, bimetallic nanocrystals have been applied and tested for many kinds of substances, such as hydrogen peroxide, uric acid, heavy metal ions, and glucose and so on. Many research groups, including us, have done some meaningful works in this field [199,200,201,202,203]. For example, PtW/MoS_2_ composite were prepared by in-situ growing on the surface of as-obtained MoS_2_ NSs (Figure 17a,b) [199]. The electrocatalytic measurements showed that PtW/MoS_2_ exhibited considerable catalytic performance towards reduction of H_2_O_2_, and the real detection limit was determined as 5 nM (Figure 17c,d). In addition, PtW/MoS_2_ also displayed good anti-interference capability (dopamine, paracetamol, NO^3−^, K^+^, hemoglobin). As shown in Figure 17e, the monodisperse AuM (M = Pd, Rh, Pt) alloyed nanoparticles were obtained with narrow size distribution and the sensing performance for H_2_O_2_ was investigated [200]. Compared with single metal nanoparticles (Au, Pd, Pt and Rh), the electrocatalytic abilities of AuM (M = Pd, Rh, Pt) bimetals to H_2_O_2_ were improved remarkably. In particular, the AuPd nanoparticles displayed an enhanced sensitivity at a relatively low test potential and good anti-interference capability to AA.

#### 4.4.3. Catalytic Combustion Gas Sensor

The catalytic combustion gas sensor is based on the thermal effect principle of catalytic combustion. It is composed of detection elements and compensation elements to form a measurement bridge. Under certain temperature conditions, the combustible gas will burn flameless on the surface of the detection element carrier and under the action of the catalyst, the carrier temperature will rise, and the platinum wire resistance inside it will rise accordingly so that the balance bridge will lose balance, and output an electrical signal proportional to the concentration of combustible gas. Among them, the doping and modification of bimetallic nanocrystals is a good solution to improve the performance of gas sensing materials, which can greatly improve their performance. Li and coworkers [204] prepared PdO/Pt-loaded mesoporous Al_2_O_3_ film for application in methane catalytic combustion. The sensing performance exhibited a short response time (<9 s) at the concentration of 10–90% LEL, and a high signal output (4.3 mV) for pre-alarm 10% LEL concentration. More importantly, the power consumption of this sensor was as low as 25 mW, which was much lower than the power of traditional sensors (~120 mW).

### 4.5. Biodetection Applications

Biodetection application of bimetals mainly utilizes some special properties of precious metals such as surface plasmon resonance (SPR) and surface-enhanced Raman scattering (SERS) [205,206]. SPR is an optical phenomenon, which applies to a collective oscillation of free electrons in the conduction band when excited by the incident light. Therefore, the SPR phenomenon can be used to track the interaction between biomolecules in natural state in real time. Plasmonic metals are mainly Au and Ag. In addition, the chemical and physical properties of bimetals are more adjustable than that of a single metal. Biosensor-based bimetallic NPs have potential applications in both research and practical uses due to better sensitivity and selectivity. SERS is a phenomenon in which Raman scattering is enhanced when the target molecules are excited in the proximity or on the surface of roughened metal substrate. In this section, we mainly introduce the state of art of biosensors based the mechanism of SPR and SERS.

#### 4.5.1. Biodetection Based on Surface Plasmon Resonance (SPR) and Local Surface Plasmon Resonance (LSPR)

The SPR biosensor is a new type of biochemical analysis system based on the principle of physical optics. It has been proved that plasmonic nanocrystals are extreme sensitivity toward changes the dielectric properties of solvent, adsorbed species [207,208]. In recent years, thanks to their well-known advantages of high sensitivity and selectivity, easy functioning, high specificity, real-time operation and fast speed, the SPR biosensor has been widely used in various fields, such as clinical diagnosis, qualitative and quantitative testing of genetically modified ingredients, virus identification, environmental microbial testing, drug screening and genetic analysis, etc. [209,210,211]. Local surface plasmon resonance (local SPR, LSPR) is a resonance phenomenon of free electrons in metals, and it occurs in metal nanostructures. The extinction phenomenon in LSPR is caused by the absorption and scattering of light by nanoparticles. LSPR has obvious advantages over traditional SPR biosensors due to the relatively simple optical system and the ability to amplify SPR response signal changes. Therefore, biosensors based on SPR and LSPR have been significantly developed in recent years.

**Figure 18:**
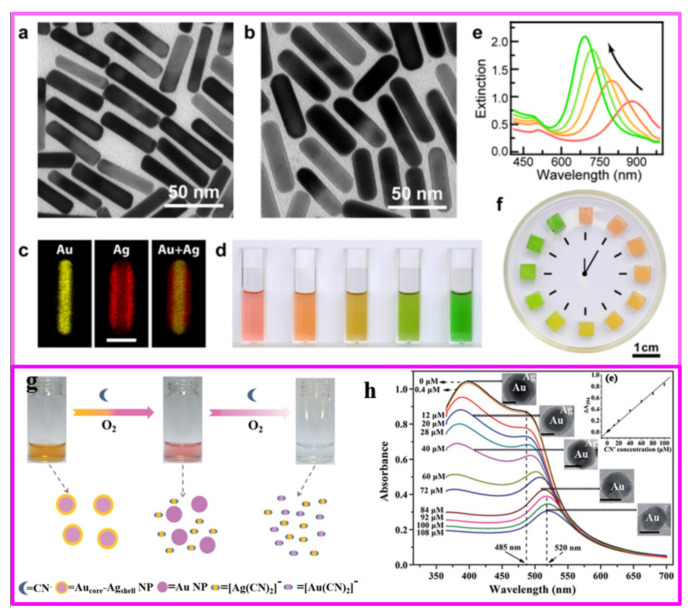
TEM images of the Au (**a**) and Au/Ag (**b**) nanorods; (**c**) Elemental mapping images of Au/Ag nanorod; (**d**) the color change during the reaction process; (**e**) Extinction spectra of the samples in (**d**); (**f**) Photograph of the hydrogel cubes. Reproduced with permission from [214]. Copyright American Chemical Society, 2013. (**g**) Schematic diagram of the Au@Ag core/shell NPs for detecting cyanide; (**h**) UV-vis spectra of the Au@Ag core/shell NPs with the addition of increasing cyanide. Reproduced with permission from [215]. Copyright Royal Society of Chemistry, 2014.

Gold and silver are the typical plasmonic metals. Ag NPs always exhibit a stronger and sharper plasmon resonance than Au NPs. However, pure silver is rarely used in practical applications due to the instability caused by moisture, acids, oxidation, heat, and light. The addition of another metal to the Ag system is a good solution to improve the stability and maintain the excellent SPR performance. Yu et al. [212] specifically combined the gold nanorods with the Fab fragments of IgG molecules to perform biological detection. When the IgG antibody molecules in the solution specifically bind onto the gold nanorods surface, it will cause the red shift of the longitudinal plasmon resonance absorption peak of the gold nanorods. Bimetallic nanocrystals have higher SPR refractive index sensitivity than that of single metal nanocrystals, and more suitable for bio-detection. For example, Ding et al. [213] prepared Ag nanoparticles onto ITO substrate by electrodeposition and then deposited a thin Au shell onto Ag surface by immersing the Ag/ITO in HAuCl_4_ solution. Compared with Ag/ITO, the obtained Ag@Au/ITO had high chemical stability and low biological toxicity, and exhibited high SPR refractive index sensitivity. In addition, subsequent antibody detection experiments showed that this modification greatly improved the detection sensitivity. In addition, the shift of SPR absorption peak caused by the structural change of bimetallic nanoparticles under the action of external molecules can also be used for biological detection. Yan etc. prepared Au@Ag bimetallic nanocrystals and used it to detect the storage time of food [214]. The continuous deposition of silver on the surface of gold nanorods will cause the shift of the SPR absorption peak. As shown in Figure 18a–f, at the initial time, the solution of Au nanorods was reddish brown. With the extension of time, silver deposited on the surface of Au nanorods, leading to the solution gradually becoming orange-yellow-green. Therefore, color change can reflect the proliferation of *E. coli* and the storage time of food.

Moreover, Au/Ag bimetallic nanocrystals are usually used as colorimetric probes for detecting cyanide. The colorimetric mechanism is that cyanide could react with Au and Ag to form a metal–cyanide complex in the presence of oxygen (Figure 18g). According to this mechanism, some colorimetric cyanide sensors have been made [215,216]. For example, Zeng and co-workers have synthesized Au@Ag core-shell nanocrystals by using citrate capped Au nanoparticles for the colorimetric cyanide sensing [215]. The results showed that UV-vis spectra of Au@Ag core-shell nanocrystals were very sensitive to the thickness of Ag shell (Figure 18h), and thus significantly sensitive to cyanide. Furthermore, the concurrent induced color changes were obvious and visible to the naked eye.

#### 4.5.2. Biodetection Based on Surface-Enhanced Raman Scattering (SERS)

**Figure 19:**
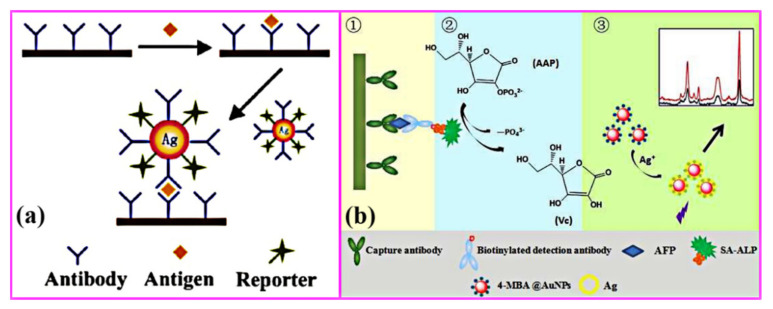
(**a**) Schematic illustration of immunoassay method based on SERS. Reproduced with permission from [217]. Copyright American Chemical Society, 2006. (**b**) Illustration of enzyme induced metallization of AFP for SERS immunoanalysis. Reproduced with permission from [221]. Copyright Royal Society of Chemistry, 2017.

SERS is a phenomenon in which Raman scattering is enhanced when molecules of interest are excited in the proximity or on the surface of roughened metal substrate. There is no consensus on the nature of enhancement mechanism, and most scholars believe that SERS enhancement is mainly composed of physical enhancement and chemical enhancement, and the former is dominant. Sliver nanocrystals often possess higher SERS enhancement due to the significant plasmonic efficiency, but the stability of Ag NPs is poor [217]. Gold nanocrystals are widely used in biological applications due to their good biocompatibility and high stability [218]. The Au–Ag bimetal has been proved to be better nanostructures than single Au or Ag due to combining the advantage of both metals. Therefore, AuAg NPs are widely used in the identification of tumor cells and normal cells, detection of tumor location and DNA diagnosis. As shown in Figure 19, the process of immunoassay using SERS includes three steps: (i) modifying the substrate for capturing antibody; (ii) SERS probe for specific antigen recognition; (iii) Raman spectrum detection. Grubisha et al. detected prostate antigen (PSA) by using gold nanoparticles as Raman signal amplification carrier, and the detection limit was as low as ~1 pg/mL [219]. However, Tian et al. found in the experiment that the highest enhancement ability of Ag@Au bimetallic nanoparticles could reach more than 10 times that of single metal (Ag or Au), which greatly improved the detection sensitivity [220]. Huang et al. also obtained a similar result [221]. They developed a novel SERS immunoassay with high sensitivity and selectivity for detecting cancer marker such as AFP. The Au@Ag core-shell structure could take advantage of the properties of both Au and Ag NPs, and improved the insufficient aspects of sensitivity and stability. In addition, with the increase of the thickness of the Ag shell, the SERS signal was increased regularly, which can be ascribed to the generally accepted assumptions that the larger the size of NPs, the more significant the SERS enhancement effect.

## 5. Conclusions and Prospects

In the past decade, great progress has been made in the shape-controlled synthesis, characterization, and applications of bimetallic nanocrystals. Introducing a metal into a noble metal system can not only save an amount of noble metal and reduce the cost of the catalyst, but improve the performance by changing the composition, controlling the atomic arrangement, and adjusting the electronic structure. Bimetallic nanocrystals often exhibit some new properties and capabilities due to the synergistic effect of bimetals, not just the simple addition of the properties associated with two single metals. Therefore, bimetallic nanocrystals have been widely used in some important fields including catalysis, sensing, and energy conversion. This article reviewed the progress in the synthesis strategy, and important applications of bimetallic nanocrystals. Four typical synthesis methods for co-reduction, seeded-growth, thermal decomposition and galvanic replacement were summarized. However, several hundred articles about the synthesis and applications of bimetallic nanocrystals have been published each year. Therefore, it is difficult for us to consider all contributions to this field, and we apologize for neglecting some important contributions.

Although there are many achievements in the controllable synthesis and applications of bimetallic nanocatalysts, further research is needed in the following aspects: precisely controlling the morphology, size and structure of bimetallic nanocrystals, and establishing the theory of nanocrystal nucleation and growth mechanisms. Although many synthetic strategies have been developed and many rich nanostructures have been obtained, traditional synthetic methods are not sufficient to control the surface structure of metal nanocrystals at the atomic level. Although researchers have been able to use some in situ characterization techniques to observe the nucleation process of nanocrystals, the process is extremely fast and complex. Therefore, it is difficult for people to understand this process in depth. In addition, the nucleation method has a great influence on the crystal plane, defects, and growth method. Therefore, there are still huge opportunities and challenges with regard to the formation mechanism of bimetallic nanocrystals.

Developing methods for scaling up production: if nanocrystals are to be used in industry, they must be made in large quantities, so exploring new methods or optimizing existing methods is a good solution to expand production. At present, the experimental devices for synthesizing bimetallic nanocrystals mainly include hydrothermal reactor three-necked flasks, etc. Their capacity generally does not exceed 200 mL. While these methods could successfully produce products at the milligram scale, it is impossible to fully meet industrial-level usage. If the volume of the reactor is simply increased, and the amount of raw materials is also proportionally increased, which will alter the reaction kinetics of the nanocrystals, the shape, structure and size of the products cannot be controlled well. In recent years, some new reaction systems have been developed, such as continuous flow and droplet-based systems, which have improved product control and reproducibility.

Chemical and thermal stability of bimetallic nanocrystals: although many published works in the literature reported that bimetals possess good thermal stability, the original shape could also transfer to another thermodynamically favorable shape or architecture by excessive heating together for a long time. In terms of chemical stability, due to the difference of the redox potential, the more reactive metal in bimetallic nanocrystals tends to leach in some applications such as fuel cells. Therefore, the thermal and chemical stability of bimetallic nanocrystals still need to be further studied in specific applications.

Although there are still some challenges, some promising work has been published in recent years. It was proved that introducing a third metal in the bimetallic system may be a promising strategy for improving the performance of bimetallic nanocrystals due to the possible synergetic effects [222]. For example, by decorating PtCu nanocrystals with Ni, a PtCu@PtCuNi catalyst showed a higher activity and stability for ORR than that of PtCu, and mass and specific activities were obviously improved in comparison to those of commercial Pt/C [223]. The method to improve the properties of bimetals is definitely not limited to the introduction of the third metal, and we should use our imagination to continue exploring this. With the in-depth research on the controlled synthesis chemistry and catalytic chemistry of bimetallic nanocrystals, we believe that we will have the ability to precisely control the composition, morphology and size of nanocatalysts, and achieve low-cost, large-scale synthesis of high-performance bimetallic nanocatalysts.

## Figures and Tables

**Table 1:** Summary of shapes that have been successfully prepared for bimetallic nanocrystals with typical morphology.

Structure	Shapes	Models	Bimetals and References
Zero-dimensional	Tetrahedron	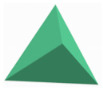	PdPt [26], PdCu [27]
	Octahedron and truncated octahedron	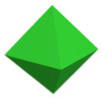 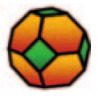	Pt@Pd [28], Pt_3_Ni [29] PtCo [30]
	Cube and truncated cube	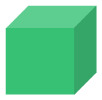 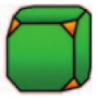	Au-Pd [31], Pt-Pd [32]
	Icosahedron	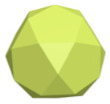	Pt-Pd [32]
	Rhombic dodecahedron	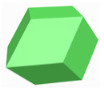	AuPd [33], NiPt [34]
One-dimensional	Nanorods	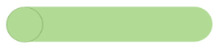	CuPt [35], Pt_3_Fe [36]
	Wavy wire	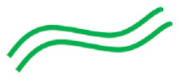	PtCo [37], PtCu [38], PdPt [39]
Two-dimensional	Triangular plates	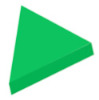	Pd@Pt [40], Ag-Pd [41]
	Hexagonal plates	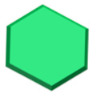	Pd@Pt [40], PdAg [42]
Hollow structure	Nanoframework	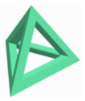 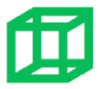	CuPd [43], PdRh [44]
	Nanocage	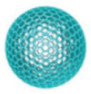 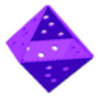 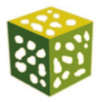	PdPt [45], PtNi [46] PdCu [47], AuAg [48]

**Table 2:** Reduction potentials of the metal ions [16].

Reduction Reaction	E_o_ (V vs. SHE) ^a^
Au^3+^ + 3e^−^ 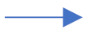 Au	1.50
Pt^2+^ + 2e^−^ 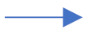 Pt	1.18
Ir^3+^ + 3e^−^ 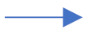 Ir	1.16
Pd^2+^ + 2e^−^ 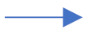 Pd	0.95
Ag^+^ + e^−^ 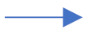 Ag	0.80
Rh^3+^ + 3e^−^ 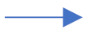 Rh	0.76
Ru^3+^ + 3e^−^ 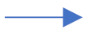 Ru	0.45
Cu^2+^ + 2e^−^ 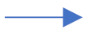 Cu	0.34
Ni^2+^ + 2e^−^ 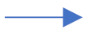 Ni	−0.25
Co^2+^ + 2e^−^ 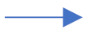 Co	−0.28
Fe^2+^ + 2e^−^ 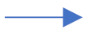 Fe	−0.44

SHE: standard hydogen electrode. ^a^ Standard condition: 25 °C and 1 atm.

**Table 3:** Advantages and disadvantages of different methods for preparing bimetallic nanocrystals.

Synthesis Method	Advantages	Disadvantages
Co-reduction	(i) Simple synthesis process (ii) Easily control composition (iii) High yield and low cost	(i) The overuse reducing agent pollutes environment
Seed-mediated growth	(i) Tunable size, shape, and composition (ii) Facile and wide applicability	(i) Complicated synthesis process (ii) Time- and cost-consuming
Thermal decomposition	(i) Facile, time-saving and highly efficient (ii) controllable composition, size and morphology	Limited metal precursors (only some organometallic precursors)
Galvanic replacement reaction	(i) Easily prepare hollow bimetallic nanostructure (ii) controllable size and morphology of Ag seed	(i) Complicated synthesis process (ii) Time- and cost-consuming

## References

[1] Parker J.F., Fields-Zinna C.A., Murray R.W. (2010). The story of a monodisperse gold nanoparticle: Au_25_L_18_. Acc. Chem. Res..

[2] El-Sayed M.A. (2004). Small is different: Shape-, size-, and composition-dependent properties of some colloidal semiconductor nanocrystals. Acc. Chem. Res..

[3] Valden M., Lai X., Goodman D.W. (1998). Onset of catalytic activity of gold clusters on titania with the appearance of nonmetallic properties. Science.

[4] Kim H.Y., Lee H.M., Henkelman G. (2012). CO oxidation mechanism on CeO_2_-supported Au nanoparticles. J. Am. Chem. Soc..

[5] Ha H., Yoon S., An K., Kim H.Y. (2018). Catalytic CO oxidation over Au nanoparticles supported on CeO_2_ nanocrystals: Effect of the Au–CeO_2_ interface. ACS Catal..

[6] Liu J.-X., Su Y., Filot I.A., Hensen E.J. (2018). A linear scaling relation for CO oxidation on CeO_2_-supported Pd. J. Am. Chem. Soc..

[7] Zhao X., Yang S., Sun Z., Cui N., Zhao P., Tang Q., Tong Y., Liu Y. (2019). Enhancing the intrinsic stretchability of micropatterned gold film by covalent linkage of carbon nanotubes for wearable electronics. ACS Appl. Electron. Mater..

[8] Murray C., Sun S., Doyle H., Betley T. (2001). Monodisperse 3d transition-metal (Co, Ni, Fe) nanoparticles and their assembly intonanoparticle superlattices. MRS Bull..

[9] Luo M., Zhao Z., Zhang Y., Sun Y., Xing Y., Lv F., Yang Y., Zhang X., Hwang S., Qin Y. (2019). PdMo bimetallene for oxygen reduction catalysis. Nature.

[10] Zhang N., Shao Q., Xiao X., Huang X. (2019). Advanced catalysts derived from composition-segregated platinum–nickel nanostructures: New opportunities and challenges. Adv. Funct. Mater..

[11] Liu M., Zhao Z., Duan X., Huang Y. (2019). Nanoscale structure design for high-performance Pt-based ORR catalysts. Adv. Mater..

[12] Bai T., Lu P., Zhang K., Zhou P., Liu Y., Guo Z., Lu X. (2017). Gold/silver bimetallic nanocrystals: Controllable synthesis and biomedical applications. J. Biomed. Nanotechnol..

[13] Wang D., Deng L., Cai H., Yang J., Bao L., Zhu Y., Wang X. (2020). Bimetallic PtCu nanocrystal sensitization WO_3_ hollow spheres for highly efficient 3-Hydroxy-2-butanone biomarker detection. ACS Appl. Mater. Interfaces.

[14] Xin H., Holewinski A., Schweitzer N., Nikolla E., Linic S. (2012). Electronic structure engineering in heterogeneous catalysis: Identifying novel alloy catalysts based on rapid screening for materials with desired electronic properties. Top. Catal..

[15] Zhao X., Xi C., Zhang R., Song L., Wang C., Spendelow J.S., Frenkel A.I., Yang J., Xin H.L., Sasaki K. (2020). High-Performance nitrogen-doped intermetallic PtNi catalyst for the oxygen reduction reaction. ACS Catal..

[16] Gilroy K.D., Ruditskiy A., Peng H.-C., Qin D., Xia Y. (2016). Bimetallic nanocrystals: Syntheses, properties, and applications. Chem. Rev..

[17] Wang D., Li Y. (2011). Bimetallic nanocrystals: Liquid-phase synthesis and catalytic applications. Adv. Mater..

[18] Kwon T., Jun M., Lee K. (2020). Catalytic nanoframes and beyond. Adv. Mater..

[19] Ferrando R., Jellinek J., Johnston R. (2008). Nanoalloys: From theory to applications of alloy clusters and nanoparticles. Chem. Rev..

[20] Gamler J.T.L., Ashberry H.M., Skrabalak S.E., Koczkur K.M. (2018). Random alloyed versus intermetallic nanoparticles: A comparison of electrocatalytic performance. Adv. Mater..

[21] Huang X., Li Y., Li Y., Zhou H., Duan X., Huang Y. (2012). Synthesis of PtPd bimetal nanocrystals with controllable shape, composition, and their tunable catalytic properties. Nano Lett..

[22] Xia Y., Xiong Y., Lim B., Skrabalak S.E. (2009). Shape-controlled synthesis of metal nanocrystals: Simple chemistry meets complex physics?. Angew. Chem. Int. Ed..

[23] Srinoi P., Chen Y.-T., Vittur V., Marquez M.D., Lee T.R. (2018). Bimetallic nanoparticles: Enhanced magnetic and optical properties for emerging biological applications. Appl. Sci..

[24] Sytwu K., Vadai M., Dionne J.A. (2019). Bimetallic nanostructures: Combining plasmonic and catalytic metals for photocatalysis. Adv. Phys. X.

[25] Min Y., Wang Y. (2020). Manipulating bimetallic nanostructures with tunable localized surface plasmon resonance and their applications for sensing. Front. Chem..

[26] Yin A., Min X., Zhang Y., Yan C. (2011). Shape-selective synthesis and facet-dependent enhanced electrocatalytic activity and durability of monodisperse sub-10 nm Pt-Pd tetrahedrons and cubes. J. Am. Chem. Soc..

[27] Zhang J., Dong Y., Liu Q., Zhou M., Mi G., Du X. (2019). Hierarchically alloyed Pd–Cu microarchitecture with tunable shapes: Morphological engineering, and catalysis for hydrogen evolution reaction of ammonia borane. Int. J. Hydrogen Energy.

[28] Habas S., Lee H., Radmilovic V., Somorjai G., Yang P. (2007). Shaping binary metal nanocrystals through epitaxial seeded growth. Nat. Mater..

[29] Zhang J., Yang H., Fang J., Zou S. (2010). Synthesis and oxygen reduction activity of shape-controlled Pt_3_Ni nanopolyhedra. Nano Lett..

[30] Shen M., Xie M., Slack J., Waldrop K., Chen Z., Lyu Z., Cao S., Zhao M., Chi M., Pintauro P.N. (2020). Pt−Co truncated octahedral nanocrystals as a highly active and durable catalyst toward the oxygen reduction reaction. Nanoscale.

[31] Fan F.-R., Liu D.-Y., Wu Y.-F., Duan S., Xie Z.-X., Jiang Z.-Y., Tian Z.-Q. (2008). Epitaxial growth of heterogeneous metal nanocrystals: From gold nano-octahedra to palladium and silver nanocubes. J. Am. Chem. Soc..

[32] Yin A.-X., Min X.-Q., Zhu W., Wu H.-S., Zhang Y.-W., Yan C.-H. (2012). Multiply twinned Pt–Pd nanoicosahedrons as highly active electrocatalysts for methanol oxidation. Chem. Commun..

[33] Lee Y.W., Kim M., Kang S.W., Han S.W. (2011). Polyhedral bimetallic alloy nanocrystals exclusively bound by {110} facets: Au–Pd rhombic dodecahedra. Angew. Chem. Int. Ed..

[34] Singh G., Sunde S., Seland F. (2020). Synthesis of CO-tolerant Ni-Pt rhombic dodecahedra bimetallic electrocatalytic nanoparticles. ChemNanoMat.

[35] Liu Q., Yan Z., Henderson N.L., Bauer J.C., Goodman D.W., Batteas J.D., Schaak R.E. (2009). Synthesis of CuPt nanorod catalysts with tunable lengths. J. Am. Chem. Soc..

[36] Liao H.G., Cui L., Whitelam S., Zheng H. (2012). Real-time imaging of Pt_3_Fe nanorod growth in solution. Science.

[37] Bao Y.X., Hao B.W., Nan L., Yan Y., Xiong W.D.L., Xin W. (2015). One-pot synthesis of Pt–Co alloy nanowire assemblies with tunable composition and enhanced electrocatalytic properties. Angew. Chem. Int. Ed..

[38] Fang D., Wan L., Jiang Q., Zhang H., Tang X., Qin X., Shao Z., Wei Z. (2019). Wavy PtCu alloy nanowire networks with abundant surface defects enhanced oxygen reduction reaction. Nano Res..

[39] Tang J.X., Chen Q.S., You L.X., Liao H.G., Sun S.G., Zhou S.G., Xu Z.N., Chen Y., Guo G.C. (2018). Screw-like PdPt nanowires as highly efficient electrocatalysts for methanol and ethylene glycol oxidation. J. Mater. Chem. A.

[40] Lim B., Lu X., Jiang M., Camargo P.H., Cho E.C., Lee E.P., Xia Y. (2008). Facile synthesis of highly faceted multioctahedral Pt nanocrystals through controlled overgrowth. Nano Lett..

[41] Lee C.-L., Tseng C.-M., Wu R.-B., Wu C.-C., Syu C.-M. (2009). Porous Ag–Pd triangle nanoplates with tunable alloy ratio for catalyzing electroless copper deposition. Colloids Surf. A Physicochem. Eng. Asp..

[42] Hu C., Mu X., Fan J., Ma H., Zhao X., Chen G., Zhou Z., Zheng N. (2016). Interfacial effects in PdAg bimetallic nanosheets for selective dehydrogenation of formic acid. ChemNanoMat.

[43] Chen Y., Yang Y., Fu G., Xu L., Sun D., Lee J.-M., Tang Y. (2018). Core–shell CuPd@Pd tetrahedra with concave structures and Pd-enriched surface boost formic acid oxidation. J. Mater. Chem. A.

[44] Xie S., Lu N., Xie Z., Wang J., Kim M.J., Xia Y. (2012). Synthesis of Pd-Rh core–frame concave nanocubes and their conversion to Rh cubic nanoframes by selective etching of the Pd cores. Angew. Chem. Int. Ed..

[45] Yin S., Xu Y., Liu S., Yu H., Wang Z., Li X., Wang L., Wang H. (2020). Binary nonmetal S and P-co-doping into mesoporous PtPd nanocages boosts oxygen reduction electrocatalysis. Nanoscale.

[46] Tian X., Zhao X., Su Y.-Q., Wang L., Wang H., Dang D., Chi B., Liu H., Hensen E.J., Lou X.W.D. (2019). Engineering bunched Pt-Ni alloy nanocages for efficient oxygen reduction in practical fuel cells. Science.

[47] Sheng J., Kang J., Ye H., Xie J., Zhao B., Fu X.-Z., Yu Y., Sun R., Wong C.-P. (2018). Porous octahedral PdCu nanocages as highly efficient electrocatalysts for the methanol oxidation reaction. J. Mater. Chem. A.

[48] Hood Z.D., Kubelick K.P., Gilroy K.D., Vanderlaan D., Yang X., Yang M., Chi M., Emelianov S.Y., Xia Y. (2019). Photothermal transformation of Au–Ag nanocages under pulsed laser irradiation. Nanoscale.

[49] Zhang L., Xie Z., Gong J. (2016). Shape-controlled synthesis of Au-Pd bimetallic nanocrystals for catalytic applications. Chem. Soc. Rev..

[50] Zhou M., Wang H., Vara M., Hood Z.D., Luo M., Yang T.H., Bao S., Chi M., Xiao P., Zhang Y. (2016). Quantitative analysis of the reduction kinetics responsible for the one-pot synthesis of Pd-Pt bimetallic nanocrystals with different structures. J. Am. Chem. Soc..

[51] Shi Y., Lyu Z., Zhao M., Chen R., Xia Y. (2020). Noble-metal nanocrystals with controlled shapes for catalytic and electrocatalytic applications. Chem. Rev..

[52] Bao Y.X., Hao B.W., Xin W., Lou X.W.D. (2012). One-pot synthesis of cubic PtCu_3_ nanocages with enhanced electrocatalytic activity for the methanol oxidation reaction. J. Am. Chem. Soc..

[53] Abe H., Matsumoto F., Alden L.R., Warren S.C., Abruna H.D., Disalvo F.J. (2008). Electrocatalytic performance of fuel oxidation by Pt_3_Ti nanoparticles. J. Am. Chem. Soc..

[54] Rong H. (2016). Kinetically controlling surface structure to construct defect-rich intermetallic nanocrystals: Effective and stable catalysts. Adv. Mater..

[55] Liu Y., Liu X., Feng Q., He D., Li Y. (2016). Intermetallic NixMy (M = Ga and Sn) nanocrystals: A non-precious metal catalyst for semi-hydrogenation of alkynes. Adv. Mater..

[56] Chung D., Jun S., Yoon G., Kwon S., Shin D., Seo P., Yoo J., Shin H., Chung Y., Kim H. (2015). Highly durable and active PtFe nanocatalyst for electrochemical oxygen reduction reaction. J. Am. Chem. Soc..

[57] Peng Z., Yang H. (2009). Designer platinum nanoparticles: Control of shape, composition in alloy, nanostructure and electrocatalytic property. Nano Today.

[58] Yan Y., Du J., Gilroy K., Yang D., Xia Y., Zhang H. (2017). Intermetallic nanocrystals: Syntheses and catalytic applications. Adv. Mater..

[59] Chi M., Wang C., Lei Y., Wang G., Li D., More K.L., Lupini A., Allard L.F., Markovic N.M., Stamenkovic V.R. (2015). Surface faceting and elemental diffusion behaviour at atomic scale for alloy nanoparticles during in situ annealing. Nat. Commun..

[60] Kang Y., Pyo J.B., Ye X., Gordon T.R., Murray C.B. (2012). Synthesis, shape control, and methanol electro-oxidation properties of Pt–Zn alloy and Pt_3_Zn intermetallic nanocrystals. ACS Nano.

[61] Wang D., Xin H., Hovden R., Wang H., Yu Y., Muller D., DiSalvo F., Abruña H.D. (2013). Structurally ordered intermetallic platinum-cobalt core-shell nanoparticles with enhanced activity and stability as oxygen reduction electrocatalysts. Nat. Mater..

[62] Ghosh Chaudhuri R., Paria S. (2012). Core/shell nanoparticles: Classes, properties, synthesis mechanisms, characterization, and applications. Chem. Rev..

[63] Zhang Y., Bu L., Jiang K., Guo S., Huang X. (2016). Concave Pd-Pt Core–shell nanocrystals with ultrathin Pt shell feature and enhanced catalytic performance. Small.

[64] Hou S., Hu X., Wen T., Liu W., Wu X. (2013). Core–shell noble metal nanostructures templated by gold nanorods. Adv. Mater..

[65] Alayoglu S., Nilekar A.U., Mavrikakis M., Eichhorn B. (2008). Ru–Pt core–shell nanoparticles for preferential oxidation of carbon monoxide in hydrogen. Nat. Mater..

[66] Choi S.I., Young A., Lee S.R., Ma C., Luo M., Chi M., Tsung C.K., Xia Y. (2019). Pd@Rh core–shell nanocrystals with well-defined facets and their enhanced catalytic performance towards CO oxidation. Nanoscale Horiz..

[67] Park J., Zhang L., Choi S.I., Roling L.T., Lu N., Herron J.A., Xie S., Wang J., Kim M.J., Mavrikakis M. (2015). Atomic layer-by-layer deposition of platinum on palladium octahedra for enhanced catalysts toward the oxygen reduction reaction. ACS Nano.

[68] Serpell C.J., Cookson J., Ozkaya D., Beer P.D. (2011). Core@shell bimetallic nanoparticle synthesis via anion coordination. Nat. Chem..

[69] Wang X., Choi S.-I., Roling L.T., Luo M., Ma C., Zhang L., Chi M., Liu J., Xie Z., Herron J.A. (2015). Palladium–platinum core-shell icosahedra with substantially enhanced activity and durability towards oxygen reduction. Nat. Commun..

[70] Sundarapandi M., Shanmugam S., Ramaraj R. (2019). Synthesis and catalytic activities of metal shells (monolayer, bilayer, and alloy layer)-coated gold octahedra toward catalytic reduction of nitroaromatics. J. Phys. Chem. C.

[71] Sneed B.T., Kuo C.-H., Brodsky C.N., Tsung C.-K. (2012). Iodide-mediated control of rhodium epitaxial growth on well-defined noble metal nanocrystals: Synthesis, characterization, and structure-dependent catalytic properties. J. Am. Chem. Soc..

[72] Zheng Z., Tachikawa T., Majima T. (2014). Single-particle study of Pt-modified Au nanorods for plasmon-enhanced hydrogen generation in visible to near-infrared region. J. Am. Chem. Soc..

[73] Gao W., Liu Q., Zhao X., Cui C., Sang Y. (2021). Electromagnetic induction effect induced high-efficiency hot charge generation and transfer in Pd-tipped Au nanorods to boost plasmon-enhanced formic acid dehydrogenation. Nano Energy.

[74] Lu N., Wang J., Xie S., Xia Y., Kim M.J. (2013). Enhanced shape stability of Pd–Rh core–frame nanocubes at elevated temperature: In situ heating transmission electron microscopy. Chem. Commun..

[75] Yoo S., Cho S., Kim D., Ih S., Lee S., Zhang L., Hao L., Jin Y.L., Liu L., Park S. (2019). 3D PtAu nanoframe superstructure as a high-performance carbon-free electrocatalyst. Nanoscale.

[76] Huang J., Mensi M., Oveisi E., Mantella V., Buonsanti R. (2019). Structural sensitivities in bimetallic catalysts for electrochemical CO_2_ reduction revealed by Ag-Cu nanodimers. J. Am. Chem. Soc..

[77] Patil R.P., Doan D., Aitken Z.H., Chen S., Gu X.W. (2020). Hardening in Au-Ag nanoboxes from stacking fault-dislocation interactions. Nat. Commun..

[78] Zhang H., Jin M., Liu H., Wang J., Kim M.J., Yang D., Xie Z., Liu J., Xia Y. (2011). Facile Synthesis of Pd–Pt Alloy Nanocages and Their Enhanced Performance for Preferential Oxidation of CO in Excess Hydrogen. ACS Nano.

[79] Sheng J., Kang J., Hu Z., Yu Y., Fu X.Z., Sun R., Wong C.P. (2018). Correction: Octahedral Pd nanocages with porous shells converted from Co(OH)_2_ nanocages with nanosheet surfaces as robust electrocatalysts for ethanol oxidation. J. Mater. Chem. A.

[80] Niu Z., Wang D., Rong Y., Peng Q., Li Y. (2012). Highly branched Pt–Ni nanocrystals enclosed by stepped surface for methanol oxidation. Chem. Sci..

[81] Park J., Wang H., Vara M., Xia Y. (2016). Platinum cubic nanoframes with enhanced catalytic activity and durability toward oxygen reduction. ChemSusChem.

[82] Ahn J., Wang D., Ding Y., Zhang J., Qin D. (2018). Site-selective carving and Co-deposition: Transformation of Ag nanocubes into concave nanocrystals encased by Au–Ag alloy frames. ACS Nano.

[83] Xiong L., Sun Z., Zhang X., Zhao L., Huang P., Chen X., Jin H., Sun H., Lian Y., Deng Z. (2019). Octahedral gold-silver nanoframes with rich crystalline defects for efficient methanol oxidation manifesting a CO-promoting effect. Nat. Commun..

[84] Mceachran M., Keogh D., Pietrobon B., Cathcart N., Gourevich I., Coombs N., Kitaev V. (2011). Ultrathin gold nanoframes through surfactant-free templating of faceted pentagonal silver nanoparticles. J. Am. Chem. Soc..

[85] Fang C., Zhao G., Zhang Z., Ding Q., Yu N., Cui Z., Bi T. (2019). AuPt bipyramid nanoframes as multifunctional platforms for in situ monitoring of the reduction of nitrobenzene and enhanced electrocatalytic methanol oxidation. Chem. A Eur. J..

[86] Nosheen F., Zhang Z-c Zhuang J., Wang X. (2013). One-pot fabrication of single-crystalline octahedral Pt–Cu nanoframes and their enhanced electrocatalytic activity. Nanoscale.

[87] Zhang Z., Luo Z., Chen B., Wei C., Zhao J., Chen J., Zhang X., Lai Z., Fan Z., Tan C. (2016). One-pot synthesis of highly anisotropic five-fold-twinned PtCu nanoframes used as a bifunctional electrocatalyst for oxygen reduction and methanol oxidation. Adv. Mater..

[88] Luo S., Shen P.K. (2016). Concave platinum–copper octopod nanoframes bounded with multiple high-index facets for efficient electrooxidation catalysis. ACS Nano.

[89] Ding J., Bu L., Guo S., Zhao Z., Zhu E., Huang Y., Huang X. (2016). Morphology and phase controlled construction of Pt–Ni nanostructures for efficient electrocatalysis. Nano Lett..

[90] Pi Y., Shao Q., Zhu X., Huang X. (2018). Dynamic structure evolution of composition segregated iridium-nickel rhombic dodecahedra toward efficient oxygen evolution electrocatalysis. ACS Nano.

[91] Yang X., Yang M., Pang B., Vara M., Xia Y. (2015). Gold nanomaterials at work in biomedicine. Chem. Rev..

[92] Piao J.-G., Wang L., Gao F., You Y.-Z., Xiong Y., Yang L. (2014). Erythrocyte membrane is an alternative coating to polyethylene glycol for prolonging the circulation lifetime of gold nanocages for photothermal therapy. ACS Nano.

[93] Li N., Wang Y., Cao W., Zhang Y., Yan T., Du B., Wei Q. (2015). An ultrasensitive electrochemical immunosensor for CEA using MWCNT-NH2 supported PdPt nanocages as labels for signal amplification. J. Mater. Chem..

[94] Sun X., Yang X., Zhang Y., Ding Y., Su D., Qin D. (2017). Pt–Ag cubic nanocages with wall thickness less than 2 nm and their enhanced catalytic activity toward oxygen reduction. Nanoscale.

[95] Zhang Y., Ahn J., Liu J., Qin D. (2018). Syntheses, plasmonic properties, and catalytic applications of Ag–Rh core-frame nanocubes and Rh nanoboxes with highly porous walls. Chem. Mater..

[96] Zhu J., Chen Z., Xie M., Lyu Z., Chi M., Mavrikakis M., Jin W., Xia Y. (2019). Iridium-based cubic nanocages with 1.1-nm-thick walls: A highly efficient and durable electrocatalyst for water oxidation in an acidic medium. Angew. Chem. Int. Ed..

[97] Oh A., Baik H., Choi D.S., Cheon J.Y., Kim B., Kim H., Kwon S.J., Joo S.H., Jung Y., Lee K. (2015). Skeletal octahedral nanoframe with cartesian coordinates via geometrically precise nanoscale phase segregation in a Pt@Ni core-shell nanocrystal. ACS Nano.

[98] Zhang Z.-P., Zhu W., Yan C.-H., Zhang Y.-W. (2015). Selective synthesis of rhodium-based nanoframe catalysts by chemical etching of 3d metals. Chem. Commun..

[99] Ham S., Jang H.J., Song Y., Shuford K.L., Park S. (2015). Octahedral and cubic gold nanoframes with platinum framework. Angew. Chem. Int. Ed..

[100] Jia Y., Jiang Y., Zhang J., Lei Z., Chen Q., Xie Z., Zheng L. (2014). Unique excavated rhombic dodecahedral PtCu_3_ alloy nanocrystals constructed with ultrathin nanosheets of high-energy {110} facets. J. Am. Chem. Soc..

[101] Chen S., Li M., Gao M., Jin J., van Spronsen M.A., Salmeron M.B., Yang P. (2020). High-performance Pt–Co nanoframes for fuel-cell electrocatalysis. Nano Lett..

[102] Lyu L.-M., Kao Y.-C., Cullen D.A., Sneed B.T., Chuang Y.-C., Kuo C.-H. (2017). Spiny rhombic dodecahedral CuPt nanoframes with enhanced catalytic performance synthesized from Cu nanocube templates. Chem. Mater..

[103] Xu L., Luo Z., Fan Z., Yu S., Chen J., Liao Y., Xue C. (2015). Controllable galvanic synthesis of triangular Ag-Pd alloy nanoframes for efficient electrocatalytic methanol oxidation. Chemistry.

[104] Park J., Kim J., Yang Y., Yoon D., Baik H., Haam S., Yang H., Lee K. (2016). RhCu 3D nanoframe as a highly active electrocatalyst for oxygen evolution reaction under alkaline condition. Adv. Sci..

[105] Huang H., Chen R., Liu M., Wang J., Kim M.J., Ye Z., Xia Y. (2020). Aqueous synthesis of Pd–M (M = Pd, Pt, and Au) decahedra with concave facets for catalytic applications. Top. Catal..

[106] Gu J., Zhang Y.W., Tao F.F. (2012). Shape control of bimetallic nanocatalysts through well-designed colloidal chemistry approaches. Chem. Soc. Rev..

[107] Lamer V.K., Dinegar R.H. (1950). Theory, production and mechanism of formation of monodispersed hydrosols. J. Am. Chem. Soc..

[108] Wu Y., Cai S., Wang D., He W., Li Y. (2012). Syntheses of water-soluble octahedral, truncated octahedral, and cubic Pt-Ni nanocrystals and their structure-activity study in model hydrogenation reactions. J. Am. Chem. Soc..

[109] Zhang Y., Han T., Fang J., Xu P., Li X., Xu J., Liu C.C. (2014). Integrated Pt_2_Ni alloy@Pt core–shell nanoarchitectures with high electrocatalytic activity for oxygen reduction reaction. J. Mater. Chem. A.

[110] Zhan F., Yin J., Zhou J., Jiao T., Zhang L., Xia M., Bai Z., Peng Q. (2020). Facile preparation and highly efficient catalytic performances of Pd-Cu bimetallic catalyst synthesized via seed-mediated method. Nanomaterials.

[111] Lu C.-L., Prasad K.S., Wu H.-L., Ho J.-A.A., Huang M.H. (2010). Au nanocube-directed fabrication of Au−Pd core−shell nanocrystals with tetrahexahedral, concave octahedral, and octahedral structures and their electrocatalytic activity. J. Am. Chem. Soc..

[112] Gao C., Lu Z., Liu Y., Zhang Q., Chi M., Cheng Q., Yin Y. (2012). Highly stable silver nanoplates for surface plasmon resonance biosensing. Angew. Chem. Int. Ed..

[113] Wang X., Vara M., Luo M., Huang H., Ruditskiy A., Park J., Bao S., Liu J., Howe J., Chi M. (2015). Pd@ Pt core–shell concave decahedra: A class of catalysts for the oxygen reduction reaction with enhanced activity and durability. J. Am. Chem. Soc..

[114] Jana N.R., Gearheart L., Murphy C.J. (2001). Evidence for seed-mediated nucleation in the chemical reduction of gold salts to gold nanoparticles. Chem. Mater..

[115] Sun Y., Yin Y., Mayers B.T., Herricks T., Xia Y. (2002). Uniform silver nanowires synthesis by reducing AgNO_3_ with ethylene glycol in the presence of seeds and poly(Vinyl Pyrrolidone). Chem. Mater..

[116] Ma Y., Li W., Cho E., Li Z., Yu T., Zeng J., Xie Z., Xia Y. (2010). Au@Ag core−shell nanocubes with finely tuned and well-controlled sizes, shell thicknesses, and optical properties. ACS Nano.

[117] Huang X., Tang S., Liu B., Ren B., Zheng N. (2011). Enhancing the photothermal stability of plasmonic metal nanoplates by a core-shell architecture. Adv. Mater..

[118] Luo N., Chen Y., Zhang D., Guo M., Xue Z., Wang X., Cheng Z., Xu J. (2020). High-sensitive MEMS hydrogen sulfide sensor made from PdRh bimetal hollow nanoframe decorated metal oxides and sensitization mechanism study. ACS Appl. Mater. Interfaces.

[119] Thomas J., Johnson B., Raja R., Sankar G., Midgley P. (2003). High-performance nanocatalysts for single-step hydrogenations. ChemInform.

[120] Ely T., Amiens C., Chaudret B., Snoeck E., Verelst M., Respaud M., Broto J. (1999). Synthesis of nickel nanoparticles. influence of aggregation induced by modification of poly(vinylpyrrolidone) chain length on their magnetic properties. Chem. Mater..

[121] Park S.J., Kim S., Lee S., Khim Z.G., Char K., Hyeon T. (2000). Synthesis and magnetic studies of uniform iron nanorods and nanospheres. J. Am. Chem. Soc..

[122] Puntes V., Krishnan K., Alivisatos A. (2001). Colloidal nanocrystal shape and size control: The case of cobalt. Science.

[123] Son S.U., Jang Y., Park J., Na H.B., Park H.M., Yun H.J., Lee J., Hyeon T. (2004). Designed synthesis of atom-economical Pd/Ni bimetallic nanoparticle-based catalysts for sonogashira coupling reactions. J. Am. Chem. Soc..

[124] Lanza R., Bersani M., Conte L., Martucci A., Canu P., Guglielmi M., Mattei G., Bello V., Centazzo M., Rosei R. (2014). Effect of crystalline phase and composition on the catalytic properties of PdSn bimetallic nanoparticles in the PROX reaction. J. Phys. Chem. C.

[125] Bönnemann H., Brand R.A., Brijoux W., Hofstadt H.W., Frerichs M., Kempter V., Maus-Friedrichs W., Matoussevitch N., Nagabhushana K.S., Voigts F. (2005). Air stable Fe and Fe-Co magnetic fluids-synthesis and characterization. Appl. Organomet. Chem..

[126] Sun Y., Mayers B.T., Xia Y. (2002). Template-engaged replacement reaction: A one-step approach to the large-scale synthesis of metal nanostructures with hollow interiors. Nano Lett..

[127] Sun Y., Wiley B., Li Z.Y., Xia Y. (2004). Synthesis and optical properties of nanorattles and multiple-walled nanoshells/nanotubes made of metal alloys. J. Am. Chem. Soc..

[128] Belousov O.V., Belousova N.V., Sirotina A.V., Solovyov L.A., Zhyzhaev A.M., Zharkov S.M., Mikhlin Y.L. (2011). Formation of bimetallic Au–Pd and Au–Pt nanoparticles under hydrothermal conditions and microwave irradiation. Langmuir.

[129] Gilroy K.D., Farzinpour P., Sundar A., Hughes R.A., Neretina S. (2014). Sacrificial templates for galvanic replacement reactions: Design criteria for the synthesis of pure Pt nanoshells with a smooth surface morphology. Chem. Mater..

[130] Pasricha R., Bala T., Biradar A.V., Umbarkar S., Sastry M. (2009). Synthesis of catalytically active porous platinum nanoparticles by transmetallation reaction and proposition of the mechanism. Small.

[131] Liang H.P., Zhang H.M., Hu J.S., Guo Y.G., Wan L.J., Bai C.L. (2004). Pt hollow nanospheres: Facile synthesis and enhanced electrocatalysts. Angew. Chem..

[132] Sun Q., Wang S., Wang R. (2012). Well-aligned CoPt hollow nanochains synthesized in water at room temperature. J. Phys. Chem. C.

[133] Mohl M., Dobo D., Kukovecz A., Konya Z., Kordas K., Wei J., Vajtai R., Ajayan P.M. (2011). Formation of CuPd and CuPt bimetallic nanotubes by galvanic replacement reaction. J. Phys. Chem. C.

[134] Mayers B., Jiang X., Sunderland D., Cattle B., Xia Y. (2003). Hollow nanostructures of platinum with controllable dimensions can be synthesized by templating against selenium nanowires and colloids. J. Am. Chem. Soc..

[135] Lin Z.-H., Chang H.-T. (2008). Preparation of gold−tellurium hybrid nanomaterials for surface-enhanced raman spectroscopy. Langmuir.

[136] Saha S., Gayen P., Wang Z., Dixit R.J., Sharma K., Basu S., Ramani V.K. (2021). Development of bimetallic PdNi electrocatalysts toward mitigation of catalyst poisoning in direct borohydride fuel cells. ACS Catal..

[137] Lv X., Wei W., Wang H., Huang B., Dai Y. (2019). Multifunctional electrocatalyst PtM with low Pt loading and high activity towards hydrogen and oxygen electrode reactions: A computational study. Appl. Catal. B Environ..

[138] Chen F., Wang D., Chen J., Ling J., Yue H., Gou L., Tang H. (2020). PtNi nanocubes-catalyzed tyramine signal amplification electrochemiluminescence sensor for nonenzymatic and ultrasensitive detection of hepatocellular carcinoma cells. Sens. Actuators B Chem..

[139] Stamenkovic V.R., Fowler B., Mun B.S., Wang G., Ross P.N., Lucas C.A., Markovic N.M. (2007). Improved oxygen reduction activity on Pt_3_Ni(111) via increased surface site availability. Science.

[140] Wang C., Chi M., Li D., Strmcnik D., Dennis V.D.V., Wang G., Komanicky V., Chang K.C., Paulikas A.P., Tripkovic D. (2011). Design and synthesis of bimetallic electrocatalyst with multilayered Pt-skin surfaces. J. Am. Chem. Soc..

[141] Swami A., Patil I., Lokanathan M., Ingavale S., Kakade B. (2020). Enhanced oxygen reduction reaction by PdPt Alloy catalyst with stabilized platinum skin. Chem. Sel..

[142] Sui S., Wang X., Zhou X., Su Y., Riffat S., Liu C. (2017). A comprehensive review of Pt electrocatalysts for the oxygen reduction reaction: Nanostructure, activity, mechanism and carbon support in PEM fuel cells. J. Mater. Chem. A.

[143] Nørskov J.K., Rossmeisl J., Logadottir A., Lindqvist L., Kitchin J.R., Bligaard T., Jónsson H. (2004). Origin of the overpotential for oxygen reduction at a fuel-cell cathode. J. Phys. Chem. B.

[144] Stamenkovic V.R., Mun B.S., Arenz M., Mayrhofer K.J.J., Lucas C.A., Wang G., Ross P.N., Markovic N.M. (2007). Trends in electrocatalysis on extended and nanoscale Pt-bimetallic alloy surfaces. Nat. Mater..

[145] Choi S.I., Xie S., Shao M., Odell J.H., Lu N., Peng H.C., Protsailo L., Guerrero S., Park J., Xia X. (2013). Synthesis and characterization of 9 nm Pt-Ni octahedra with a record high activity of 3.3 A/mg(Pt) for the oxygen reduction reaction. Nano Lett..

[146] Wu J., Zhang J., Peng Z., Yang S., Yang H. (2010). Truncated octahedral Pt_(3)_Ni oxygen reduction reaction electrocatalysts. J. Am. Chem. Soc..

[147] Wu J., Qi L., You H., Gross A., Li J., Yang H. (2012). Icosahedral platinum alloy nanocrystals with enhanced electrocatalytic activities. J. Am. Chem. Soc..

[148] Cui C., Gan L., Heggen M. (2013). Compositional segregation in shaped Pt alloy nanoparticles and their structural behaviour during electrocatalysis. Nat. Mater..

[149] Becknell N., Kang Y., Chen C., Resasco J., Yang P. (2015). Atomic structure of Pt_3_Ni nanoframe electrocatalysts by in situ X-ray absorption spectroscopy. J. Am. Chem. Soc..

[150] Wang C., Zhang L., Yang H., Pan J., Liu J., Dotse C., Luan Y., Gao R., Lin C., Zhang J. (2017). High-indexed Pt_3_Ni alloy tetrahexahedral nanoframes evolved through preferential CO etching. Nano Lett..

[151] Luo S., Tang M., Shen P.K., Ye S. (2017). Atomic-scale preparation of octopod nanoframes with high-index facets as highly active and stable catalysts. Adv. Mater..

[152] Kwon H., Kabiraz M.K., Park J., Oh A., Baik H., Choi S.-I., Lee K. (2018). Dendrite-embedded platinum–nickel multiframes as highly active and durable electrocatalyst toward the oxygen reduction reaction. Nano Lett..

[153] Chen D., Tong Y. (2015). Irrelevance of carbon monoxide poisoning in the methanol oxidation reaction on a PtRu electrocatalyst. Angew. Chem..

[154] Chen D.J., Sun S.G., Tong Y.Y.J. (2014). On the chemistry of activating commercial carbon-supported PtRu electrocatalyst for methanol oxidation reaction. Chem. Commun..

[155] Xu H., Shang H., Wang C., Du Y. (2021). Recent progress of ultrathin 2D Pd-based nanomaterials for fuel cell electrocatalysis. Small.

[156] Xiong Y., Ma Y., Li J., Huang J., Yan Y., Zhang H., Wu J., Yang D. (2017). Strain-induced Stranski–Krastanov growth of Pd@Pt core–shell hexapods and octapods as electrocatalysts for methanol oxidation. Nanoscale.

[157] Zhang Y., Janyasupab M., Liu C.W., Li X., Xu J., Liu C.C. (2012). Three dimensional PtRh alloy porous nanostructures: Tuning the atomic composition and controlling the morphology for the application of direct methanol fuel cells. Adv. Funct. Mater..

[158] Wang L., Nemoto Y., Yamauchi Y. (2011). Direct synthesis of spatially-controlled Pt-on-Pd bimetallic nanodendrites with superior electrocatalytic activity. J. Am. Chem. Soc..

[159] Wang L., Yamauchi Y. (2013). Metallic nanocages: Synthesis of bimetallic Pt-Pd hollow nanoparticles with dendritic shells by selective chemical etching. J. Am. Chem. Soc..

[160] Li H.H., Zhao S., Gong M., Cui C.H., He D., Liang H.W., Wu L., Yu S.H. (2013). Ultrathin PtPdTe nanowires as superior catalysts for methanol electrooxidation. Angew. Chem. Int. Ed..

[161] Guo S., Zhang S., Sun X., Sun S. (2011). Synthesis of ultrathin FePtPd nanowires and their use as catalysts for methanol oxidation reaction. J. Am. Chem. Soc..

[162] Zhai Y., Zhu Z., Lu X., Zhou Z., Shao J., Zhou H.S. (2017). Facile synthesis of three-dimensional PtPdNi fused nanoarchitecture as highly active and durable electrocatalyst for methanol oxidation. ACS Appl. Energy Mater..

[163] Li H., Pan Y., Zhang D., Han Y., Wang Z., Qin Y., Lin S., Wu X., Zhao H., Lai J. (2020). Surface oxygen-mediated ultrathin PtRuM (Ni, Fe, and Co) nanowires boosting methanol oxidation reaction. J. Mater. Chem. A.

[164] Andrews S.P., Stepan A.F., Tanaka H., Ley S.V., Smith M.D. (2005). Heterogeneous or homogeneous? A case study involving palladium-containing perovskites in the suzuki reaction. Adv. Synth. Catal..

[165] Yi J., Lee W.H., Choi C.H., Lee Y., Park K.S., Min B.K., Hwang Y.J., Oh H.-S. (2019). Effect of Pt introduced on Ru-based electrocatalyst for oxygen evolution activity and stability. Electrochem. Commun..

[166] Zhou M., Bao S., Bard A.J. (2019). Probing size and substrate effects on the hydrogen evolution reaction by single isolated Pt atoms, atomic clusters, and nanoparticles. J. Am. Chem. Soc..

[167] Danilovic N., Subbaraman R., Chang K.C., Chang S.H., Kang Y., Snyder J., Paulikas A.P., Strmcnik D., Kim Y.T., Myers D. (2014). Using surface segregation to design stable Ru-Ir oxides for the oxygen evolution reaction in acidic environments. Angew. Chem..

[168] Chang S.H., Danilovic N., Chang K.-C., Subbaraman R., Paulikas A.P., Fong D.D., Highland M.J., Baldo P.M., Stamenkovic V.R., Freeland J.W. (2014). Functional links between stability and reactivity of strontium ruthenate single crystals during oxygen evolution. Nat. Commun..

[169] Shi L., Chen H., Liang X., Liu Y., Zou X. (2020). Theoretical insights into nonprecious oxygen-evolution active sites in Ti–Ir-Based perovskite solid solution electrocatalysts. J. Mater. Chem. A.

[170] Jin H., Hong Y., Yoon J., Oh A., Chaudhari N.K., Baik H., Joo S.H., Lee K. (2017). Lanthanide metal-assisted synthesis of rhombic dodecahedral MNi (M= Ir and Pt) nanoframes toward efficient oxygen evolution catalysis. Nano Energy.

[171] Pei J., Mao J., Liang X., Chen C., Peng Q., Wang D., Li Y. (2016). Ir–Cu nanoframes: One-pot synthesis and efficient electrocatalysts for oxygen evolution reaction. Chem. Commun..

[172] Pi Y., Guo J., Shao Q., Huang X. (2018). Highly efficient acidic oxygen evolution electrocatalysis enabled by porous Ir–Cu nanocrystals with three-dimensional electrocatalytic surfaces. Chem. Mater..

[173] Chen Z., Zhao H., Zhang J., Xu J. (2017). IrNi nanoparticle-decorated flower-shaped NiCo_2_O_4_ nanostructures: Controllable synthesis and enhanced electrochemical activity for oxygen evolution reaction. Sci. China Mater..

[174] Wu C.H., Liu C., Su D., Xin H.L., Fang H.-T., Eren B., Zhang S., Murray C.B., Salmeron M.B. (2019). Bimetallic synergy in cobalt–palladium nanocatalysts for CO oxidation. Nat. Catal..

[175] Miyamura H., Suzuki A., Yasukawa T., Kobayashi S. (2018). Polysilane-immobilized Rh–Pt bimetallic nanoparticles as powerful arene hydrogenation catalysts: Synthesis, reactions under batch and flow conditions and reaction mechanism. J. Am. Chem. Soc..

[176] Wang X., He Y., Liu Y., Park J., Liang X. (2018). Atomic layer deposited Pt-Co bimetallic catalysts for selective hydrogenation of α, β-unsaturated aldehydes to unsaturated alcohols. J. Catal..

[177] Taylor M.J., Beaumont S.K., Islam M.J., Tsatsos S., Parlett C.A., Issacs M.A., Kyriakou G. (2020). Atom efficient PtCu bimetallic catalysts and ultra dilute alloys for the selective hydrogenation of furfural. Appl. Catal. B Environ..

[178] Zhang Q., Zhu C., Yang G., Sun Y., Wang D., Liu J. (2019). High-performance microstructured Au-Ag bimetallic catalyst for oxidative coupling of methanol to methyl formate. Catal. Commun..

[179] Xiao Y., Varma A. (2018). Highly selective nonoxidative coupling of methane over Pt-Bi bimetallic catalysts. ACS Catal..

[180] Ba Q., Jia X., Huang L., Li X., Chen W., Mao L. (2019). Alloyed PdNi hollow nanoparticles as cocatalyst of CdS for improved photocatalytic activity toward hydrogen production. Int. J. Hydrogen Energy.

[181] Jiang Y., Wu X., Yan Y., Luo S., Li X., Huang J., Zhang H., Yang D. (2019). Coupling PtNi ultrathin nanowires with MXenes for boosting electrocatalytic hydrogen evolution in both acidic and alkaline solutions. Small.

[182] Wei R., Chen Z., Lv H., Zheng X., Ge X., Sun L., Song K., Kong C., Zhang W., Liu B. (2021). Ultrafine RhNi nanocatalysts confined in hollow mesoporous carbons for a highly efficient hydrogen production from ammonia borane. Inorg. Chem..

[183] Zhao Z., Liu H., Gao W., Xue W., Liu Z., Huang J., Pan X., Huang Y. (2018). Surface-Engineered PtNi-O nanostructure with record-high performance for electrocatalytic hydrogen evolution reaction. J. Am. Chem. Soc..

[184] Pi Y., Shao Q., Wang P., Guo J., Huang X. (2017). General Formation of Monodisperse IrM (M = Ni, Co, Fe) Bimetallic nanoclusters as bifunctional electrocatalysts for acidic overall water splitting. Adv. Funct. Mater..

[185] Huynh T.-T., Huang W.-H., Tsai M.-C., Nugraha M., Haw S.-C., Lee J.-F., Su W.-N., Hwang B.J. (2021). Synergistic hybrid support comprising TiO_2_–carbon and ordered PdNi alloy for direct hydrogen peroxide synthesis. ACS Catal..

[186] Fan A., Qin C., Zhang X., Yang J., Ge J., Wang S., Yuan X., Wang S., Dai X. (2019). Engineering FeNi alloy nanoparticles via synergistic ultralow Pt doping and nanocarbon capsulation for efficient hydrogen evolution. J. Mater. Chem. A.

[187] Li Z., Wang X., Tian W., Meng A., Yang L. (2019). CoNi bimetal cocatalyst modifying a hierarchical ZnIn_2_S_4_ nanosheet-based microsphere noble-metal-free photocatalyst for efficient visible-light-driven photocatalytic hydrogen production. ACS Sustain. Chem. Eng..

[188] Chen H.-Y., Niu H.-J., Han Z., Feng J.-J., Huang H., Wang A.-J. (2020). Simple fabrication of trimetallic platinum-nickel-cobalt hollow alloyed 3D multipods for highly boosted hydrogen evolution reaction. J. Colloid Interface Sci..

[189] Chen J., Yang Y., Su J., Jiang P., Xia G., Chen Q. (2017). Enhanced activity for hydrogen evolution reaction over CoFe catalysts by alloying with small amount of Pt. ACS Appl. Mater. Interfaces.

[190] Ozawa F., Kubo A., Hayashi T. (1992). Generation of tertiary phosphine-coordinated Pd(0) species from Pd(OAc)_2_ in the catalytic heck reaction. Chem. Lett..

[191] Beletskaya I.P., Cheprakov A.V. (2000). The heck reaction as a sharpening stone of palladium catalysis. Chem. Rev..

[192] Yin L., Liebscher J. (2007). Carbon−carbon coupling reactions catalyzed by heterogeneous palladium catalysts. Chem. Rev..

[193] Xia J., Fu Y., He G., Sun X., Wang X. (2017). Core-shell-like Ni-Pd nanoparticles supported on carbon black as a magnetically separable catalyst for green Suzuki-Miyaura coupling reactions. Appl. Catal. B Environ..

[194] Hahn C., Abram D.N., Hansen H.A., Hatsukade T., Jackson A., Johnson N.C., Hellstern T.R., Kuhl K.P., Cave E.R., Feaster J.T. (2015). Synthesis of thin film AuPd alloys and their investigation for electrocatalytic CO_2_ reduction. J. Mater. Chem. A.

[195] Jedidi A., Rasul S., Masih D., Cavallo L., Takanabe K. (2015). Generation of Cu–In alloy surfaces from CuInO_2_ as selective catalytic sites for CO_2_ electroreduction. J. Mater. Chem. A.

[196] Takashima T., Suzuki T., Irie H. (2019). Electrochemical reduction of carbon dioxide to formate on palladium-copper alloy nanoparticulate electrode. Electrochemistry.

[197] Li G., Cheng Z., Xiang Q., Yan L., Wang X., Xu J. (2019). Bimetal PdAu decorated SnO_2_ nanosheets based gas sensor with temperature-dependent dual selectivity for detecting formaldehyde and acetone. Sens. Actuators B Chem..

[198] Li G., Wang X., Yan L., Wang Y., Zhang Z., Xu J. (2019). PdPt bimetal-functionalized SnO_2_ nanosheets: Controllable synthesis and its dual selectivity for detection of carbon monoxide and methane. ACS Appl. Mater. Interfaces.

[199] Zhu L., Zhang Y., Xu P., Wen W., Li X., Xu J. (2016). PtW/MoS_2_ hybrid nanocomposite for electrochemical sensing of H_2_O_2_ released from living cells. Biosens. Bioelectron..

[200] Han T., Zhang Y., Xu J., Dong J., Liu C.-C. (2015). Monodisperse AuM (M = Pd, Rh, Pt) bimetallic nanocrystals for enhanced electrochemical detection of H_2_O_2_. Sens. Actuators B Chem..

[201] Chen W., Liu Y., Zhang Y., Fang J., Xu P., Xu J., Li X., Liu C.-C., Wen W. (2017). Highly effective and specific way for the trace analysis of carbaryl insecticides based on Au_42_Rh_58_ alloy nanocrystals. J. Mater. Chem. A.

[202] Ma L., Zhou L., He Y., Wang L., Huang Z., Jiang Y., Gao J. (2018). Hierarchical nanocomposites with an N-doped carbon shell and bimetal core: Novel enzyme nanocarriers for electrochemical pesticide detection. Biosens. Bioelectron..

[203] Xu X., Ji D., Zhang Y., Gao X., Xu P., Li X., Liu C.-C., Wen W. (2019). Detection of phenylketonuria markers using a ZIF-67 encapsulated PtPd alloy nanoparticle (PtPd@ ZIF-67)-based disposable electrochemical microsensor. ACS Appl. Mater. Interfaces.

[204] Li L., Li G., Yuan Y. (2014). Mesoporous PdO/Pt/Al_2_O_3_ film produced by reverse-micro-emulsion and its application for methane micro-sensor. RSC Adv..

[205] Zeng S., Baillargeat D., Ho H.P., Yong K.T. (2014). Nanomaterials enhanced surface plasmon resonance for biological and chemical sensing applications. Chem. Soc. Rev..

[206] Stewart M., Anderton C., Thompson L., Maria J., Gray S., Rogers J., Nuzzo R. (2008). Nanostructured plasmonic sensors. Chem. Rev..

[207] Haes A.J., Van Duyne R.P. (2002). A nanoscale optical biosensor: Sensitivity and selectivity of an approach based on the localized surface plasmon resonance spectroscopy of triangular silver nanoparticles. J. Am. Chem. Soc..

[208] Jain P.K., Qian W., El-Sayed M.A. (2006). Ultrafast cooling of photoexcited electrons in gold nanoparticle-thiolated DNA conjugates involves the dissociation of the gold-thiol bond. J. Am. Chem. Soc..

[209] Yonzon C.R., Stuart D.A., Zhang X., Mcfarland A.D., Haynes C.L., Duyne R.P.V. (2005). Towards advanced chemical and biological nanosensors—An overview. Talanta.

[210] Petryayeva E., Krull U.J. (2011). Localized surface plasmon resonance: Nanostructures, bioassays and biosensing—A review. Anal. Chim. Acta.

[211] Arsalani S., Ghodselahi T., Neishaboorynejad T., Baffa O. (2019). DNA detection based on localized surface plasmon resonance spectroscopy of Ag@Au biocomposite nanoparticles. Plasmonics.

[212] Yu C., Irudayaraj J. (2007). Multiplex Biosensor Using Gold Nanorods. Anal. Chem..

[213] Dong P., Lin Y., Deng J., Di J. (2013). Ultrathin gold-shell coated silver nanoparticles onto a glass platform for improvement of plasmonic sensors. Acs Appl. Mater. Interfaces.

[214] Zhang C., Yin A.X., Jiang R., Rong J., Yan C.H. (2013). Time-temperature indicator for perishable products based on kinetically programmable Ag overgrowth on Au nanorods. ACS Nano.

[215] Zeng J., Cao Y., Chen J., Wang X., Yu J., Yu B., Yan Z., Chen X. (2014). Au@Ag core/shell nanoparticles as colorimetric probes for cyanide sensing. Nanoscale.

[216] Li Y., Wang Q., Zhou X., Wen C.Y., Yu J., Han X., Li X., Yan Z.F., Zeng J. (2016). A convenient colorimetric method for sensitive and specific detection of cyanide using Ag@Au core–shell nanoparticles. Sens. Actuators B Chem..

[217] Jiang R., Chen H., Lei S., Qian L., Wang J. (2012). Unraveling the evolution and nature of the plasmons in (Au Core)-(Ag Shell) nanorods. Adv. Mater..

[218] Samal A.K., Polavarapu L., Rodal-Cedeira S., Liz-Marzán L.M., Pérez-Juste J., Pastoriza-Santos I. (2013). Size Tunable Au@Ag core–shell nanoparticles: Synthesis and surface-enhanced raman scattering properties. Langmuir.

[219] Grubisha D.S., Lipert R.J., Park H.Y., Driskell J., Porter M.D. (2003). Femtomolar detection of prostate-specific antigen: An immunoassay based on surface-enhanced raman scattering and immunogold labels. Anal. Chem..

[220] Cui Y., Ren B., Yao J.L., Gu R.A., Tian Z.Q. (2006). Synthesis of Ag core-Au shell bimetallic nanoparticles for immunoassay based on surface-enhanced Raman spectroscopy. J. Phys. Chem. B.

[221] Yang L., Gao M.X., Zhan L., Gong M., Zhen S.J., Huang C.Z. (2017). An enzyme-induced Au@Ag core–shell nanoStructure used for an ultrasensitive surface-enhanced Raman scattering immunoassay of cancer biomarkers. Nanoscale.

[222] Shao M., Chang Q., Dodelet J.-P., Chenitz R. (2016). Recent advances in electrocatalysts for oxygen reduction reaction. Chem. Rev..

[223] Park J., Kanti Kabiraz M., Kwon H., Park S., Baik H., Choi S.-I., Lee K. (2017). Radially phase segregated PtCu@ PtCuNi dendrite@ frame nanocatalyst for the oxygen reduction reaction. ACS Nano.

